# Predicting Knee Joint Contact Force Peaks During Gait Using a Video Camera or Wearable Sensors

**DOI:** 10.1007/s10439-024-03594-x

**Published:** 2024-08-03

**Authors:** Jere Lavikainen, Lauri Stenroth, Paavo Vartiainen, Tine Alkjær, Pasi A. Karjalainen, Marius Henriksen, Rami K. Korhonen, Mimmi Liukkonen, Mika E. Mononen

**Affiliations:** 1 Department of Technical Physics, University of Eastern Finland, Kuopio, Finland; 2 Diagnostic Imaging Centre, Kuopio University Hospital, Kuopio, Finland; 3 Department of Biomedical Sciences, University of Copenhagen, Copenhagen, Denmark; 4 The Parker Institute, Bispebjerg and Frederiksberg Hospital, Copenhagen, Denmark

**Keywords:** Knee joint, Contact force, Gait analysis, Artificial neural networks, OpenPose, Inertial measurement units, Computer vision

## Abstract

**Purpose:**

Estimating loading of the knee joint may be helpful in managing degenerative joint diseases. Contemporary methods to estimate loading involve calculating knee joint contact forces using musculoskeletal modeling and simulation from motion capture (MOCAP) data, which must be collected in a specialized environment and analyzed by a trained expert. To make the estimation of knee joint loading more accessible, simple input predictors should be used for predicting knee joint loading using artificial neural networks.

**Methods:**

We trained feedforward artificial neural networks (ANNs) to predict knee joint loading peaks from the mass, height, age, sex, walking speed, and knee flexion angle (KFA) of subjects using their existing MOCAP data. We also collected an independent MOCAP dataset while recording walking with a video camera (VC) and inertial measurement units (IMUs). We quantified the prediction accuracy of the ANNs using walking speed and KFA estimates from (1) MOCAP data, (2) VC data, and (3) IMU data separately (i.e., we quantified three sets of prediction accuracy metrics).

**Results:**

Using portable modalities, we achieved prediction accuracies between 0.13 and 0.37 root mean square error normalized to the mean of the musculoskeletal analysis-based reference values. The correlation between the predicted and reference loading peaks varied between 0.65 and 0.91. This was comparable to the prediction accuracies obtained when obtaining predictors from motion capture data.

**Discussion:**

The prediction results show that both VCs and IMUs can be used to estimate predictors that can be used in estimating knee joint loading outside the motion laboratory. Future studies should investigate the usability of the methods in an out-of-laboratory setting.

**Supplementary Information:**

The online version contains supplementary material available at 10.1007/s10439-024-03594-x.

## Introduction

It is believed that excess joint loading may have adverse effects on joint health and affect the development of joint diseases like knee osteoarthritis (KOA) [11, 17, 19]. Existing literature is inconclusive on how knee joint loading exactly affects KOA [18], but it is nonetheless believed to play a part at least in the structural progression of the disease [3, 5, 11, 17, 24]. Structural degeneration of cartilage is likely irreversible and therefore, prevention would be the best treatment to fight its progression. Assuming there is causation between knee joint loading and diseases linked to structural cartilage degeneration, developing accessible methods to estimate knee joint loading is extremely important. This would allow the design of better individualized preventive methods to reduce the incidence of such diseases.

Personalized knee joint loading can be estimated with musculoskeletal simulation and modeling, where the contact forces in the joint are calculated [13]. The input data required for these calculations is cumbersome to collect, as it requires a separate motion laboratory with expensive motion capture (MOCAP) equipment and time-consuming subject preparation. Therefore, easier ways to calculate knee joint contact forces (KJCFs) are required to enable biomechanically informed knee joint loading estimates in clinical settings, where they could be used for guiding rehabilitation and predicting the onset of degenerative joint diseases. In the recent years, machine learning techniques, especially feedforward artificial neural networks (ANNs) have been used for predicting knee joint loading [2, 5, 16, 29, 33]. However, to calculate the predictions, these studies require MOCAP data collected in a motion laboratory. While MOCAP data is extensive and enables high-accuracy prediction models, it limits the usability of the methods in out-of-laboratory settings where effortless data collection is emphasized.

Inertial measurement units (IMUs) and video cameras (VCs) provide a more portable and effortless alternative to MOCAP and could be utilized in out-of-laboratory (e.g., clinical) settings because of their ease of use. IMUs can provide wireless information about orientations and accelerations of body segments when they are strapped to the subject [30]. VC data, e.g., from a webcam, can be analyzed to identify anatomical landmarks of the subject using human pose estimation solutions [7, 26]. Therefore, both IMUs and VCs represent possible modalities for obtaining biomechanically relevant input data that could be used (e.g., combined with demographic information) for predicting KJCFs in a clinical setting.

IMU and VC data have already been used for estimating KJCFs with machine learning. For instance, Raimondo et al. calculated KJCFs using ground reaction forces and moments estimated from IMU data [27]. Stetter et al. trained and evaluated ANNs to predict KJCFs from acceleration and angular velocity data measured with IMUs on the thigh and the shank [33]. Uhlrich et al. developed the OpenCap platform that can solve KJCFs from smartphone videos [35]. While most existing methods eliminate the need for MOCAP data, they are designed for specific measurement technologies. Prediction of KJCFs would be more modular, and thus more accessible in out-of-laboratory use, if it was designed to only require input data that is readily available in clinics (e.g., demographic information like mass, height, age, and sex) or obtainable from different portable modalities (such as VC and IMUs). These modular methods to predict KJCFs should be designed to utilize inputs that are so simple that any of several available technologies, rather than a specific one, could be used for obtaining them.

In our previous study [22], we explored the possibility of using ANNs to predict knee joint loading peaks from simple input variables alias predictors and quantified the accuracy of the predictions. There, we chose mass, height, age, sex, walking speed and knee frontal plane alignment as predictors. These predictors were chosen because they can be collected (mass, height, age, sex) or estimated (walking speed, knee alignment) without MOCAP data. In that study, the predictors were nonetheless calculated from MOCAP data. Because our long-term goal is to bring biomechanical assessment of knee joint loading peaks out of the motion laboratory to, e.g., clinician’s appointment, the need for MOCAP data should be eliminated in the future.

Therefore, the aim of this study is (1) to develop and evaluate methods to estimate simple predictors of KJCF without MOCAP data and (2) to demonstrate the accuracy of ANNs trained on MOCAP data to predict KJCF peaks from those predictors. We hypothesized that ANNs trained on MOCAP data can with sufficient accuracy predict knee joint contact force peaks during gait using the participants’ mass, height, age, sex, walking speed, and knee flexion angle (KFA) as the predictors, where the latter two are estimated from IMU and VC data. We quantified the accuracy of estimating walking speed and KFA, and the accuracy of utilizing those predictors to predict KJCF peaks with ANNs, by comparing the predictor estimates and KJCF predictions to ground truth values obtained from MOCAP and musculoskeletal modeling and simulation for IMU and VC data separately.

## Materials and Methods

**Fig. 1:**
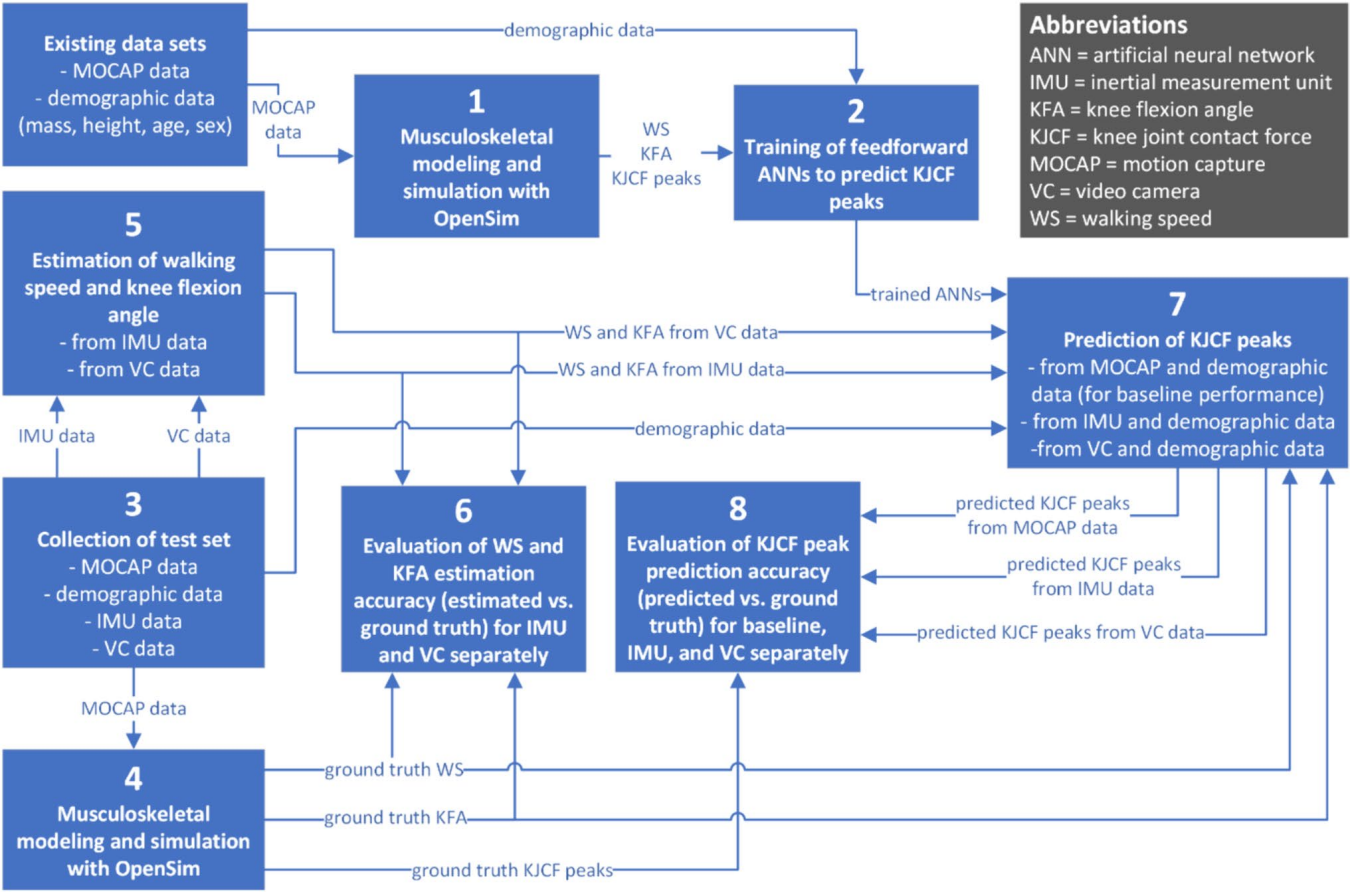
Overview of the study. Motion capture data from existing datasets was analyzed (1) using OpenSim and used for training feedforward artificial neural networks to predict knee joint contact force peaks from the mass, height, age, sex, walking speed, and knee flexion angle predictors of subjects (2). An independent dataset was collected (3) and analyzed (4). Walking speed and knee flexion angle predictors were then estimated from video camera and IMU data separately from the independent dataset (5) and compared against ground truth values from analyzed MOCAP data (6). Finally, the trained networks were used for predicting knee joint contact force peaks (7), and the predictions were compared to ground truth values calculated from the test set using musculoskeletal modeling and simulation (8)

This work comprised the following main steps (Fig. 1):

1. Musculoskeletal analysis of existing MOCAP datasets [1, 6, 15, 20, 32]
2. Training of ANNs to predict KJCF peaks using the existing musculoskeletal analyzed data
3. Collection of an independent dataset comprising MOCAP, IMU, and VC data
4. Musculoskeletal analysis of the collected dataset to obtain ground truth walking speed, KFA predictor and KJCF peaks
5. Estimation of walking speed and KFA from (a) IMU and (b) VC data
6. Evaluation of estimation accuracy of walking speed and KFA predictor from (a) IMU and (b) VC data
7. Prediction of KJCF peaks using demographic data and walking speed and KFA predictors from (a) IMU and (b) VC data
8. Evaluation of prediction accuracy of the trained ANNs against the independent dataset.

### Musculoskeletal Analysis of Existing MOCAP Datasets (Fig. 1: Step 1)

Five existing MOCAP datasets were used: four open datasets of healthy participants [6, 15, 20, 32] and one dataset of KOA patients [1] (a total of 290 subjects as described in our previous study, where demographic information per dataset is also presented [22]). All datasets contained marker trajectories and ground reaction forces and moments (GRF) from overground walking trials. We used the musculoskeletal simulation and modeling software OpenSim 4.3 [8] for analyzing the data in a multi-stage workflow, which involved:

i. Scaling a musculoskeletal model to subject-specific dimensions and muscle strengths
ii. Calculating the kinematics of motion from marker trajectories using the inverse kinematics tool
iii. Calculating the dynamics of motion from force plate data and the kinematics using the inverse dynamics tool
iv. Estimating muscle activations using the static optimization tool
v. Finally, estimating joint reaction forces, i.e., knee joint contact forces based on data from the previous stages (i-iv). The analysis was done identically to our previous study [22], which contains a more detailed explanation. In short, we incorporated a bicompartmental knee mechanism [23] into a full-body musculoskeletal model [28] to calculate bicompartmental joint reaction forces applied to the medial and lateral contact points of the tibia; the extracted KJCFs then comprised the full-stance time series of the medial compartment, lateral compartment, and summed (medial + lateral) compressive tibiofemoral contact forces.

**Fig. 2:**
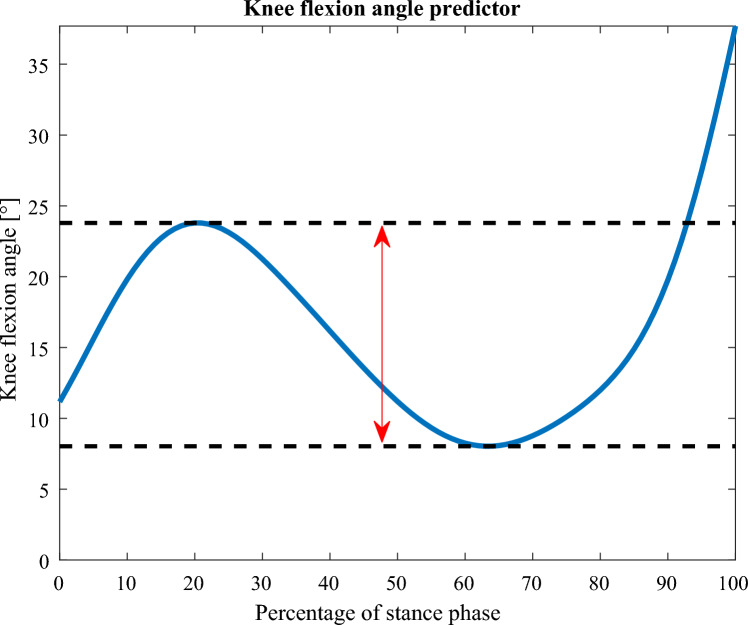
An illustration of the knee flexion angle (KFA) predictor. The knee flexion curve during stance is shown in blue. The KFA predictor is calculated in degrees as the difference between the maximum of the first half of the flexion curve and the minimum of the second half of the flexion curve. The red double-ended arrow shows the maximum and the minimum used for calculating the KFA predictor

**Fig. 3:**
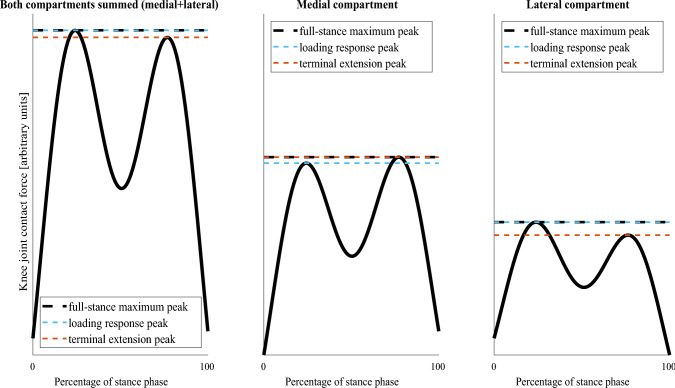
An illustration of the extracted knee joint contact force peaks. We extracted a total of nine peaks: loading response (1st peak in time series), terminal extension (2nd peak in time series), and full-stance maximum over the sum of compartmental loadings (left), medial compartmental loading (center), and lateral compartmental loading (right). The full-stance maximum peak always equals either the loading response peak or the terminal extension peak, whichever is higher. The illustrated loading curves are artificially generated

From the output of the musculoskeletal analysis, the trajectories of markers on the pelvis, time series of KFAs, and time series of KJCFs were further analyzed. We calculated walking speed from the trajectories of markers on the pelvis cluster. For KFAs, we constructed a KFA predictor by calculating the difference between the maximum of the first half and the minimum of the second half of the stance phase (Fig. 2). For KJCFs, which were characterized by two distinct peaks during the stance phase (loading response and terminal extension), we identified the maximum of each peak. The maximum loading of the full stance phase was also identified. Using the peak detection method described in our previous study [22], all these three peak values were identified separately for medial and lateral compartments and their combined load; this resulted in nine different KJCF peaks (Fig. 3), i.e., our response variables.

### Training of ANNs Using the Existing Musculoskeletal Analyzed Data (Fig. 1: Step 2)

We trained shallow feedforward ANNs to predict KJCF peaks. The predictors were the subjects’ mass, height, age, sex, walking speed, and KFA predictor. Unlike in our previous study [22], frontal knee alignment was replaced with KFA because we had no reliable method to measure the frontal alignment without MOCAP data; furthermore, we assumed that KFA correlates with some KJCF peaks because KFA has been shown to correlate with peak external knee extension moments on KOA patients [14]. A separate ANN was trained for each KJCF peak, resulting in nine ANNs that each predicted a different peak from the KJCF time series during the stance phase of gait.

We used MATLAB R2023a for training the ANNs. Each network had a single hidden layer with just one hidden node. We trained the networks with the musculoskeletal analyzed data retrieved from existing MOCAP datasets [1, 6, 15, 20, 32]. Because one of the datasets contained KOA patients [1], we first used that dataset to pre-train the network and then used the remaining four datasets [6, 15, 20, 32] to finish the training. Therefore, the training dataset comprised 290 subjects and approximately 5000 walking trials (the exact number of included trials varied with the response variable [22]). No separate validation subset was split from the training data because we used a backpropagation training algorithm with Bayesian regularization to avoid overfitting. Detailed information about the design and training of the networks is presented in the Supplementary material. The performance of the trained networks was later evaluated against the test subset (see **step 8** for details).

### Collection of an Independent Walking Dataset (Fig. 1: Step 3)

The study received approval from the University of Eastern Finland Ethics Committee and adheres to the Declaration of Helsinki. We recruited and analyzed 51 healthy volunteers without knee pain or abnormalities in their walking to participate in walking measurements in a motion laboratory. Due to issues with automatic labeling of experimental marker data that could not be fixed without great effort, 5 were excluded and the analyzed set of participants consisted of 46 participants. These participants (29 male, 17 female) were between 20 and 45 years of age (mean ± standard deviation: 29 ± 6), had body mass indices between 18.8 and 40.4 (25.1 ± 4.2), and performed overground walking trials with three instructed velocities: comfortable walking speed, 25% slower than comfortable (referred to as “slow”), and 25% faster than comfortable (referred to as “fast”). The demographics between our training and test sets do not fully match and are compared in the Supplementary material (Supplementary discussion, “Demographic observations in the training and test sets”). Before walking trials, we measured the participants’ mass, height, and inter-ASIS distance (i.e., the distance between the palpated left and the right anterior superior iliac spine protrusions on the ilium) and collected their age and sex with a survey. The dominant leg was also determined by this survey as the leg the participants would prefer for kicking a football and all 46 participants identified their right leg as the dominant leg; we later analyzed this “dominant leg” to retrieve KFAs and KJCFs (Fig. 1: steps 5 and 4, respectively). We chose to limit our analysis on only the dominant leg to ensure no differences in kinematics or kinetics between the dominant and the non-dominant leg affect the results of the study.

**Fig. 4:**
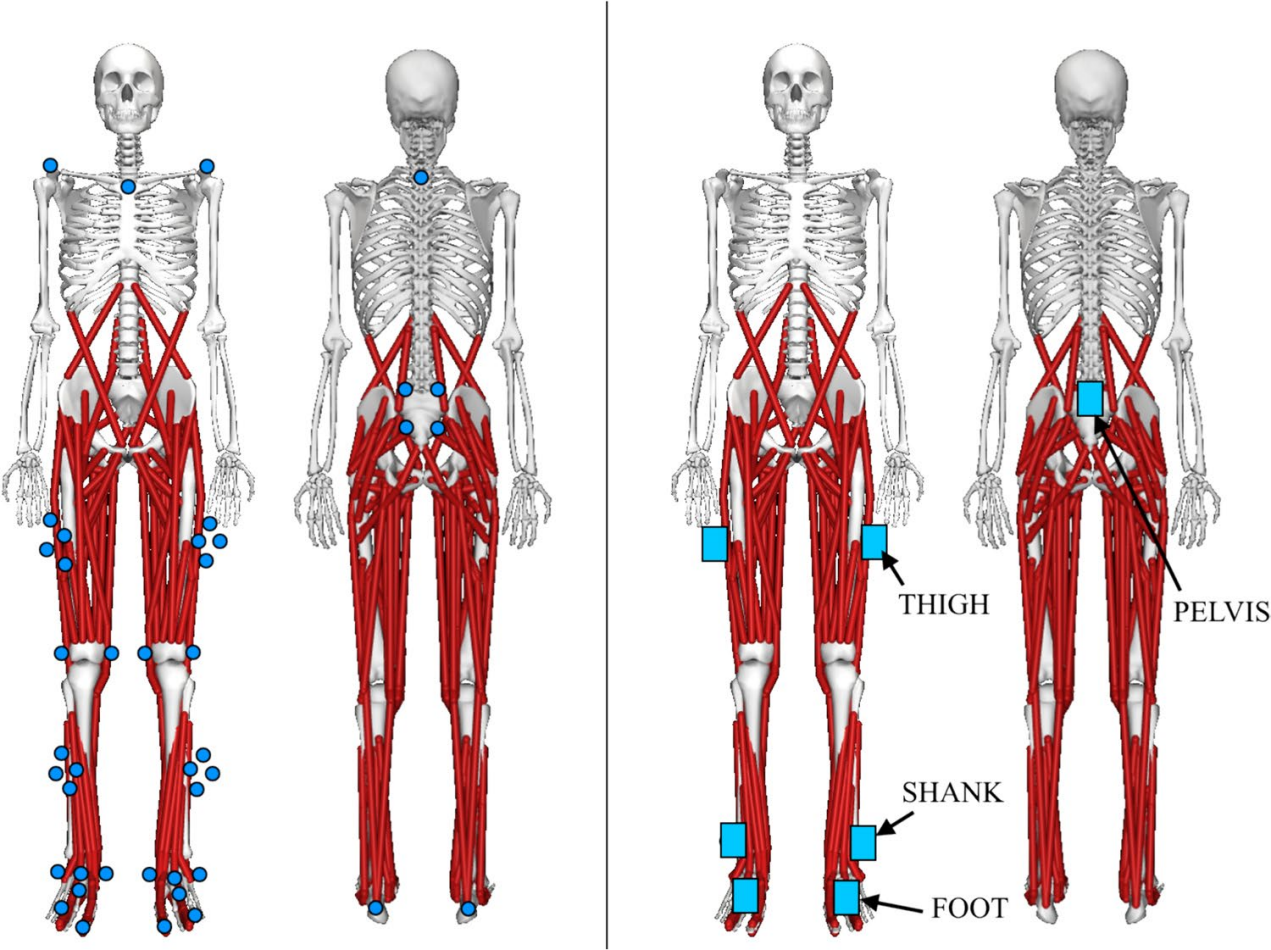
Placement of markers (left) and inertial measurement units (right) on the participants

We equipped the participants with 42 reflective markers that were tracked with a motion analysis system (10 Vicon Vero cameras, Vicon Nexus software, Vicon Motion Systems Ltd, UK) and 7 nine-degree-of-freedom IMUs (Xsens MTw Awinda, Movella Inc, Henderson, NV, USA). The participants had a marker cluster with 4 markers each on the pelvis, both thighs, and both shanks. On both feet, we placed individual markers on the 1st distal phalanx, 4th proximal phalanx, and posterior to the heel. Individual markers were also placed on medial and lateral malleoli on both ankles, and medial and lateral epicondyles on both knees. Additionally, we placed individual markers on the manubrium of the sternum, on acromia, and on the 7th cervical vertebra; we placed these upper-body markers on clothing instead of skin if any risk of marker occlusion by clothing was present. Behind the pelvis and on thighs, the IMUs were placed on a slot in the marker clusters; on shanks, we strapped the IMUs on the lateral side just above the ankle; on feet, we taped the IMUs on the metatarsals. Finally, two markers were placed on each metatarsal IMU, one posteriorly and the other anteriorly. An illustration of the marker and IMU placement is provided in Fig. 4.

For each instructed velocity (slow, comfortable, and fast), the participants performed 10 overground walking trials in one direction and another 10 in the opposite direction. A video camera (VICON Vue) placed 4 m from the walking line recorded sagittal-plane video at a 1280x720 resolution. Marker, IMU and VC data were collected at 100 Hz. We also collected GRFs of the foot of the dominant leg from a floor-embedded force plate (AMTI OR6-7) at 1000 Hz.

Additionally, we scanned the right knee of each participant using low-field magnetic resonance imaging (MRI) and measured the intercondylar distance from the frontal and transverse views of the 3D reconstruction of the knee. We defined intercondylar distance as the distance between the mid-points of lateral and femoral condyles. This distance was used only to set the intercondylar distance of the knee of the musculoskeletal models in the test set (Fig. 1: step 4). Note that the use of MRI data to set the intercondylar distance of the musculoskeletal model is not relevant for the prediction accuracy of the neural networks (Fig. 1: step 8) nor required to use the trained networks.

In conclusion, we collected demographic information and measured a total of 60 walking trials per participant. For each trial, we captured marker trajectories and GRFs, IMU acceleration and orientation data, and sagittal-plane video of the participant. We present videos demonstrating the walking trials in the Supplementary videos. The collected pseudonymized raw data is available on Zenodo (https://zenodo.org/records/10559504).

### Musculoskeletal Analysis of the Collected Dataset (Fig. 1: Step 4)

The independent MOCAP dataset that we collected underwent a musculoskeletal modeling and analysis pipeline identical to the pipeline we described for existing datasets from literature (Fig. 1: step 1), with model scaling-related exceptions mentioned below:

1. We used the SCoRE [9] and SARA [10] protocols to find the hip and knee joint centers and knee axes of rotation because they have been shown to locate hip joint centers and knee axes of rotation accurately and repeatably [34]. This information was used for scaling the femurs during model scaling. However, because SCoRE has been shown to exaggerate the lateral position of the hip joint center [4], we chose not to use it for scaling the pelvis because it would have made the pelvis too large. Instead, we scaled the pelvis according to the previously collected (see earlier steps) manual measurement of the participant’s inter-ASIS distance. For setting the intercondylar distance the knee joint, we used the intercondylar distance measured from low-field MRI of the right knee.
2. We scaled the maximum isometric muscle forces of the musculoskeletal models according to $1.5\times {(\frac{\text{SUBJECT MASS}}{\text{GENERIC MODEL MASS}})}^{2/3}$. The factor of 1.5 was used to prevent maximal muscle activations during the fastest walking speeds (the value chosen was based on our experience rather than literature). The exponential factor of 2/3 comes from assuming an allometric relationship between body mass and cross-sectional area of muscle (which determines muscle force) [12].
3. For determining the neutral static standing pose, we used the SCoRE-estimated hip joint centers and knee joint centers, markers on both sides of the ankles, and markers of the foot projected to the floor. The floor-projected foot markers let us set the orientation of the foot during standing as the foot was flat on the ground. Furthermore, we assumed that pelvis orientation was neutral while standing (no pelvic tilt or list).

We extracted walking speed, KFA, and KJCF peaks like we described earlier for existing MOCAP datasets. These KJCF peaks were later used as ground truth values when evaluating the trained ANNs (Fig. 1: step 8).

### KFA and Walking Speed from IMU and VC Data (Fig. 1: Step 5)

In addition to MOCAP data, the collected walking dataset contained data from two portable modalities: a sagittal-plane VC and IMUs. To estimate KFA and walking speed from both modalities, we analyzed them separately.

We analyzed the sagittal-plane video data by running the OpenPose computer vision algorithm [7] at a 480 × 272 resolution to detect anatomical landmarks, i.e., keypoints, of the participant in each image frame. We estimated walking speed by associating pixels in the image to real world displacement and tracking the horizontal displacement of the hip keypoint in the image. We estimated the time series of KFA by calculating the angle between vectors defined using hip, knee, and ankle keypoints, and then extracted the KFA predictor from the resulting time series of estimated KFA.

For IMU data, we estimated walking speed using orientation data from thigh and shank IMUs. We used acceleration data for identifying gait cycles and calculate the gait period, i.e., the duration of each gait cycle. We assumed that thighs and shanks are rigid bodies whose lengths are given as a fraction of the participant’s height and that the left and the right thigh bodies connect at the proximal end. We could then use the 3D orientation of the IMUs for rotating corresponding thighs and shanks and calculate the 3D positions of the distal ends of the shank bodies. Finally, we calculated stride length based on the positions of the distal ends of the shanks and divided the stride lengths by the gait period to obtain an estimate of walking speed. For KFA, we assumed that the axis of greatest rotation during walking was the axis rotating objects in the sagittal plane for the thigh and shank IMUs. We then used principal component analysis on orientation data of thigh and shank IMUs to find the rotation in the axis of greatest rotation, set the rotation to zero when the participant stood in neutral position in the first frame of the trial, and calculated the angle between the principal axes of thigh and shank IMUs during the trial. This method resulted in an estimate of the time series of knee flexion angle, from which we extracted the KFA predictor. More detailed information about IMU and VC-based methods is presented in the Supplementary material.

Finally, to quantify the accuracy of determining the KFA predictor and walking speed from VC and IMU data, we compared their estimates to the ground truth values obtained from the MOCAP and musculoskeletal analysis of the walking dataset we had collected (Fig. 1: step 6).

### Evaluation of Prediction Accuracy of Trained Artificial Neural Networks (Fig. 1: Step 8)

The walking dataset we collected formed an independent test subset for evaluating KJCF peak prediction accuracy of trained ANNs. We considered the KJCF peaks from the musculoskeletal analysis pipeline as ground truth values. The demographic information (mass, height, age, sex) and walking speed and KFA (estimated from IMUs and VCs separately) from the collected walking dataset were fed into the ANNs to obtain the predicted KJCF peaks (Fig. 1: step 7). Additionally, we input the demographic information together with MOCAP-based walking speed and KFA to retrieve baseline KJCF peak predictions. The KJCF peak predictions obtained using IMU and VC data were compared against the baseline predictions to evaluate how much modality-specific error in walking speed and KFA affected the predicted KJCF peaks.

We only included valid trials for the independent test set. Trial validity was determined by visually inspecting the time series of ground reaction forces and hip and knee joint angles for anomalies that indicated, e.g., that the participant did not step fully on the force plate (see Supplementary material for further details). For each walking configuration (combination of instructed velocity and direction), we only included participants with at least five valid trials. If a participant had less than five valid walking trials in any walking configuration (i.e., less than half of all trials for a configuration), the participant was eliminated (i.e., all trials of that participant were omitted from subsequent analysis). This resulted in the elimination of two to six participants depending on modality (IMU or VC) and response variable (different peaks of the KJCF time series).

For those participants eligible for the test set, we picked the first five valid trials per walking configuration in the test set to evaluate the prediction accuracy of KJCF peaks. This criterion ensured that the test set was balanced in the sense that no participant or walking configuration was emphasized over the others. Therefore, the KJCF prediction results presented in this paper include 30 walking trials per participant (six different walking configurations with five included trials per configuration). Additionally, results for different walking speeds are presented in Supplementary material. To evaluate how beneficial the inclusion of the KFA predictor or walking speed in the predictor set is, we also trained neural networks with only demographic predictors (mass, height, age, sex), demographic predictors and the KFA predictor, demographic predictors and walking speed, and the full predictor set of demographic predictors, the KFA predictor, and walking speed.

### Comparison Between Artificial Neural Networks and Multiple Linear Regression

To compare the ANN-based prediction models of KJCF peaks against a more intuitively understandable approach, we fitted first-order polynomial multiple linear regression (MLR) models to predict the response variables. We used the ordinary least squares method to fit MLR models to equation

$$y=a\times \text{mass}+b\times \text{height}+c\times \text{age}+d\times \text{sex}+e\times \text{walking speed}+f\times \text{KFA predictor}+g,$$

where coefficients *a* through *g* are the fitted parameters and *y* is the prediction. One MLR model was fitted for each response variable. For fitting the MLR models, we used the training data of the ANNs, and for evaluating the performance of the MLR models, we used the test data of the ANNs. We used the same data for ANN and MLR models to enable comparison between them.

### Statistical Tests

We conducted analysis of variance (ANOVA) tests to evaluate if occluding the analyzed leg, using different predictor sets, or using MLR prediction models instead of ANN prediction models have a statistically significant effect on the accuracy of predicting KJCF peaks. The level of statistical significance was set at *p* = 0.05, where values at or below the level were interpreted as statistically significant.

For testing if occluding the analyzed leg (as determined by walking direction) had a statistically significant effect on KJCF peak prediction accuracy, we used a two-way repeated measures ANOVA test. Within-participant means of relative change in mean absolute error from baseline were used as the observations of the dependent variable. Walking direction (“left”, where the left leg was facing the camera and occluded the analyzed right leg; or “right”, where the analyzed leg was visible to the camera), and response variable (nine loading peaks) were used as the two within-subjects factors.

A three-way repeated measures ANOVA test was used to determine if the predictor set had a significant effect on KJCF peak prediction accuracy. Within-participant normalized root-mean square errors of KJCF peak prediction were used as the observations of the dependent variable. The predictor set (demographic variables only, demographic + KFA, demographic + walking speed, or demographic + walking speed + KFA), modality (baseline, IMU, or VC) and response variable (nine loading peaks) were used as the three within-subjects factors. Because there were four instead of two different predictor sets, we used a multiple pairwise comparison procedure with Bonferroni correction to test for statistically significant differences between paired predictor sets.

Finally, a three-way repeated measures ANOVA test was used to determine if there were significant differences in KJCF peak prediction accuracy between ANN and MLR models. Within-participant normalized root-mean square errors of KJCF peak prediction were used as the observations of the dependent variable. Prediction method (ANN or MLR), modality (baseline, IMU, or VC) and response variable (nine loading peaks) were used as the three within-subjects factors.

## Results

The dispersion of the walking speed and KFA predictors in the collected dataset shows greater relative variability for the KFA predictor than for walking speed (Table 1). We estimated walking speed from IMU data with marginally greater RMSE and smaller Pearson correlation coefficient (calculated against MOCAP-based “ground truth” values) than from VC data (Table 2). However, the estimated KFA predictor from IMU data had a clearly smaller RMSE and greater Pearson correlation coefficient than from VC data. Furthermore, compared to IMU data, estimation from VC data showed greater inter-subject variation in both metrics for both predictors. Relative to the mean of the reference values (Table 1), the RMSEs presented in Table 2 correspond to mean relative errors of 4.8% for walking speed and 23.0% for the KFA predictor.

**Table 1:** Mean, standard deviation (STD), range, minimum, and maximum of motion capture-based reference walking speed and KFA predictor in the collected dataset

Predictor	Mean ± STD	Range (min–max)
walking speed (cm/s)	133.56 ± 26.68	180.12 (65.53–245.65)
KFA predictor (°)	16.30 ± 5.64	36.72 (1.60–38.33)

The values are calculated over all successful walking trials in the collected dataset (*N* = 2614) without grouping by participant

**Table 2:** Root-mean-square errors (RMSE) and Pearson correlation coefficients (*R*) and their standard deviations (STD) of estimated walking speed and KFA predictor

Predictor	IMU		VC	
	RMSE ± STD	*R* ± STD	RMSE ± STD	*R* ± STD
walking speed (cm/s)	6.52 ± 4.42	0.95 ± 0.08	6.29 ± 9.16	0.96 ± 0.10
KFA predictor (°)	3.75 ± 1.54	0.91 ± 0.11	5.24 ± 1.83	0.58 ± 0.24

The RMSE and *R* are calculated as inter-subject means where each participant is equally weighted and STD from intra-subject means of RMSE and *R*. The metrics are presented when estimating the predictors from IMU or VC data separately. The metrics were calculated by comparing the IMU- and VC-based values against corresponding ground truth values obtained from the musculoskeletal simulation pipeline that utilized motion capture data

Between baseline, IMU-based, and VC-based predictions, the KJCF peak prediction errors vary little, and no method clearly outperforms the others in terms of prediction accuracy (Table 3). However, for terminal extension peaks, the IMU-based predictions correlate better with the musculoskeletal modeling and simulation-based peaks than the VC-based predictions do. In all three cases, the correlation between predicted and ground truth values was higher for summed peaks than for compartmental peaks. For IMU and VC, loading response peaks were predicted with the highest NRMSE. Furthermore, in terms of NRMSE, loading peaks in the lateral compartment were always predicted less accurately than medial or summed loading peaks.

**Table 3:** Root-mean-square errors (RMSE), RMSEs normalized to the mean of the ground truth values (NRMSE), and Pearson correlation coefficients (*R*) between loading peaks predicted with ANNs and ground truth loading peaks from a musculoskeletal simulation and modeling pipeline

Response variable	Baseline			IMU			VC		
	RMSE (*N*)	NRMSE	*R*	RMSE (*N*)	NMRSE	*R*	RMSE (*N*)	NRMSE	*R*
Full-stance max (summed)	407	0.15	0.89	418	0.15	0.89	373	0.14	0.91
Full-stance max (medial)	248	0.14	0.87	237	0.13	0.87	255	0.14	0.85
Full-stance max (lateral)	329	0.32	0.82	329	0.32	0.80	291	0.28	0.84
Loading response (summed)	472	0.18	0.90	565	0.22	0.89	520	0.20	0.90
loading response (medial)	281	0.17	0.81	298	0.18	0.81	294	0.18	0.81
Loading response (lateral)	310	0.31	0.89	375	0.37	0.87	354	0.35	0.88
Terminal extension (summed)	389	0.16	0.81	382	0.16	0.82	391	0.16	0.80
Terminal extension (medial)	319	0.19	0.72	306	0.18	0.73	325	0.19	0.69
Terminal extension (lateral)	214	0.27	0.66	214	0.27	0.66	217	0.28	0.65

The prediction accuracy metrics are presented for nine different response variables and three methods of obtaining walking speed and knee flexion angle predictors (“baseline” from MOCAP-based data, “IMU” from IMU-based data, and “VC” from video camera-based data). To enable comparability, values shown comprise participants that were valid for all modality and response variable combinations, totaling 37 participants. The metrics were calculated by comparing the predicted values against corresponding ground truth values obtained from the musculoskeletal simulation pipeline that utilized motion capture data

**Fig. 5:**
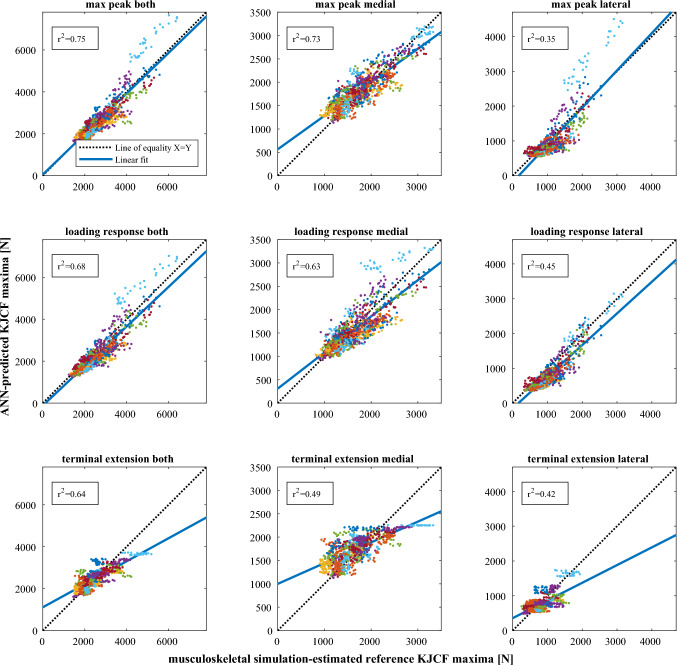
Accuracies of predicting KJCF peaks with ANNs when utilizing walking speed and knee flexion angle from motion capture (baseline) data. Each point represents a KJCF peak from one walking trial, calculated with musculoskeletal simulation and modeling (*x* axis) and ANNs (*y* axis). Walking trials from different subjects have different colors. All plots use the same participants and walking trials

The linear fits to baseline predictions versus reference peaks in Fig. 5 indicate that in general ANN prediction is biased towards too high KJCF peaks at low levels of loading and too low KJCF peaks at high levels of loading. This effect is emphasized when predicting terminal extension peaks. Similar figures for IMU and VC data-based predictions are presented in the Supplementary material.

**Fig. 6:**
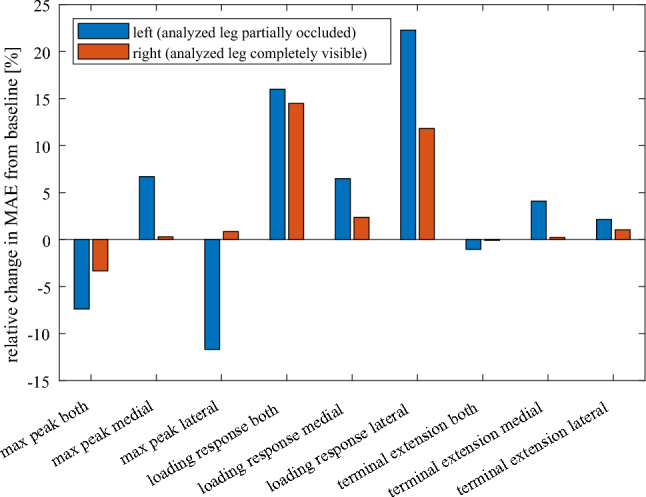
The effect of walking direction on the change (from baseline) of mean absolute error (MAE) of predicting knee joint contact force peaks when walking speed and KFA predictor are estimated from video camera data. “Left” indicates that the analyzed leg (right leg) was occluded to the camera by the left leg, and “right” indicates that the analyzed leg was visible to the camera and occluded the left leg

Walking direction, determining whether the analyzed leg was completely visible to the camera or partially occluded by the other leg, was visually observed to have no impact on KJCF prediction accuracy when estimating walking speed and KFA predictor from VC data compared to estimating them from MOCAP data (Fig. 6). In accordance with visual observations, a two-way repeated measures ANOVA test found no statistically significant effect between walking direction and the relative change in mean absolute error from baseline (*p* = 0.07).

**Fig. 7:**
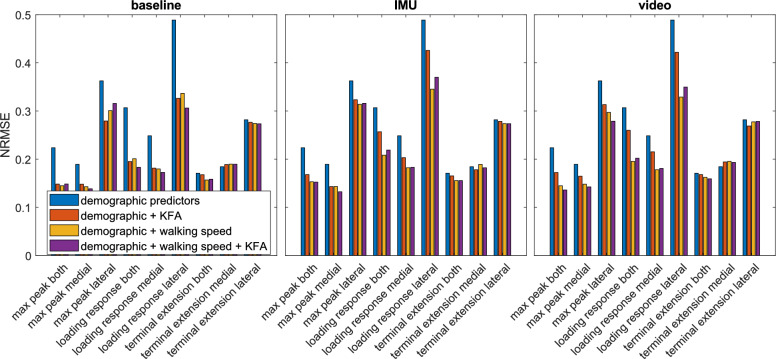
Comparison of NRMSE errors between ANN-predicted peaks and "ground truth" peaks from MS analysis for different predictor sets when walking speed and KFA predictor are from motion capture (left, baseline), inertial measurement unit (center, IMU), or video camera (right, video) data. Blue bars represent error when only demographic predictors (mass, height, age, and sex) are in the set; red bars when the predictor set comprises the demographic predictors and KFA predictor; orange bars when the predictor set comprises the demographic predictors and walking speed; and purple bars with the full predictor set. The values shown comprise participants that were valid for all modality and response variable combinations, totaling 37 participants

Including either the KFA predictor or walking speed in the predictor set decreases the error of predicting KJCF peaks compared with using only demographic predictors (Fig. 7). However, the inclusion of walking speed appears to decrease the error more than the inclusion of the KFA predictor. The error of predicting full-stance peaks (max peak both/medial/lateral) further seems to decrease slightly when the KFA predictor is included in addition to demographic predictors and walking speed, but its inclusion has negligible effect on the error or even increases it slightly for other response variables.

A three-way repeated measures ANOVA test showed that the choice of the predictor set has a significant effect on prediction error (*p* < 0.001). Prediction errors differ significantly when using only demographic predictors compared to using any of the other three predictor sets (*p* < 0.02). Furthermore, including walking speed in the predictor set in addition to demographic variables and the KFA predictor has a significant effect on prediction error (*p* = 0.02). However, no significant effect on prediction error was observed when comparing the predictor set with demographic variables and the KFA predictor against the predictor set with demographic variables and walking speed (*p* = 0.53), nor when including the KFA predictor in the predictor set when it already contained demographic variables and walking speed (*p* = 1.00).

**Fig. 8:**
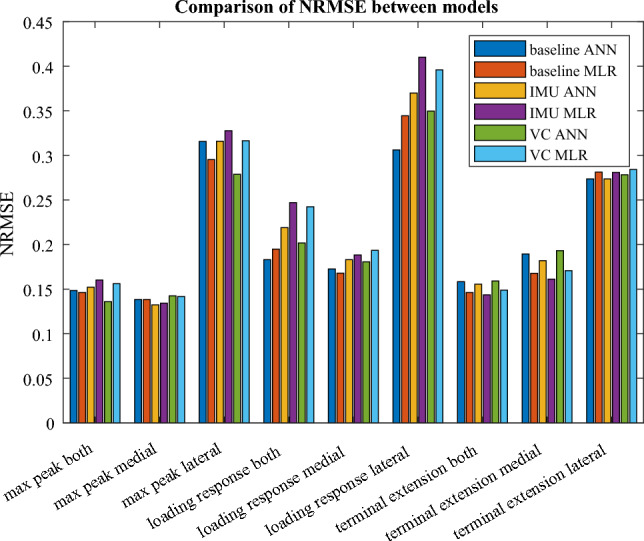
Comparison of prediction RMSE normalized to the mean of the reference values (NMRSE) between artificial neural network (ANN) models and first-order polynomial multiple linear regression (MLR) models. The errors are grouped by response variable. In each group, prediction NRMSE is shown for predictions using walking speed and KFA predictor from motion capture data (baseline), inertial measurement unit data (IMU), and video camera data (VC)

Comparison of prediction NRMSE between trained ANN models and fitted first-order polynomial multiple linear regression models show that the difference in prediction NRMSE varies depending on the response variable and whether the predictions used walking speed and KFA predictors estimated from MOCAP data, IMU data, or VC data (Fig. 8). ANN models outperformed MLR models for lateral loading response peaks and lateral terminal extension peaks, MLR models outperformed ANN models for summed and medial terminal extension peaks, and otherwise, neither type of model consistently outperformed the other. However, a three-way repeated measures ANOVA test found no statistically significant effect on prediction error between the methods (ANN versus MLR, *p* = 0.94).

## Discussion

Our main findings show that predicting knee joint contact force peaks using demographic data with walking speed and KFA predictor from IMU or VC data separately yields similar accuracies to using predictors from MOCAP data. However, prediction accuracy varied depending on the peak of the KJCF time series, with lower errors for summed and medial loading peaks than for lateral loading peaks.

Using portable modalities to estimate walking speed and KFA predictor did not meaningfully affect the prediction accuracy of KJCF peaks (Table 3) despite the errors in estimating the predictors (Table 2). In some cases, using portable modalities even improved accuracy; in these cases, the error in walking speed and KFA predictor likely shifted the prediction towards the reference value by coincidence. Because the KFA predictor was estimated with greater error than walking speed (Table 2), we are satisfied that it had little effect on KJCF prediction accuracy when walking speed was already present in the predictor set (Fig. 7). Therefore, estimating KFA may be unnecessary to reach the prediction accuracies reported in this study. Furthermore, the measurement of walking speed could be done with even simpler modalities (such as a stopwatch) than IMUs and VCs, so if we dropped the KFA predictor, we could perhaps make our approach even more portable without compromising much prediction accuracy.

Our ANN-based KJCF peak predictions are particularly inaccurate for one participant (Fig. 5, teal points forming the clusters of highest reference loading). This participant was notably heavy, representing the highest BMI in the test set (40.4 compared to the second-highest 34.8). This observation implies that the ANN prediction models cannot accurately predict loading peaks for very obese individuals, which is expected given that the obese individuals in the training dataset came mostly from the KOA patient dataset, where loading values are unlikely to represent non-pathological yet obese loading, and which was only used in pre-training the networks. Therefore, including more non-pathological but obese subjects in the training set could improve the capability of the network to predict their loading peaks. It is also possible that our musculoskeletal model scaling parameters are unsuitable for very heavy subjects, which could cause distorted loading peaks in the training data, but we consider it unlikely because we have validated our musculoskeletal simulation pipeline [22] and scaled muscle strength of the models according to body mass.

When estimating KFA from VC data, we expected the occlusion of the analyzed leg to adversely affect the detection of the keypoints using OpenPose and result in greater error in KJCF peaks compared to baseline, where KFA was calculated from MOCAP data. Because walking direction (determining whether the analyzed leg was visible or occluded) had no unambiguous effect on the prediction accuracy of KJCF peaks (Fig. 6), KFA estimation remained largely unhindered by the occlusion of the analyzed leg. This is a promising observation because partial occlusion of the subject is a drawback of VC-based motion analysis methods; reliable tracking of the occluded leg would benefit out-of-laboratory use, where the operator may lack experience in preventing occlusions or correcting occlusion-related errors after the measurement.

No definitive superiority in terms of minimized NRMSE exists between ANN and MLR models (Fig. 8). Because the differences between them are consistently noticeable when predicting lateral loading response peaks, the relationships between predictors and the response for that response variable may have more nonlinearity than relationships for response variables do. Consequently, the nonlinear approximation capability of ANNs, which first-order polynomial MLR models lack, could result in superior ANN performance. Similarly, summed and medial terminal extension peaks may have comparably more linear relationships between the predictors and the response, which is why linear MLR models predict them with lower error than nonlinear ANN models do. If so, then ANNs may be a better option for predicting loading peaks that are mostly monotonous (as lateral peaks sometimes were); it is also possible that lateral compartmental loading lacks the double bump shape that is usually present in the medial compartmental loading profile and which we assumed to exist in both compartments. Future studies should investigate the capability of higher-order polynomial and regularized models to predict KJCF peaks, as those models are easier for others to adopt and provide better information about the inference (i.e., the relationship between the input and the output) of the model than ANN-based models do.

We demonstrated the estimation of the KFA predictor and walking speed with two portable modalities: a limited set of IMUs and a single video camera. However, our method to train neural network models for predicting KJCF peaks is independent of these two modalities because the models are trained with MOCAP data. Therefore, the trained models could be used with any modality that can estimate the KFA predictor and walking speed. Observations related to estimating the predictors using IMU and VC data are presented in more detail in Supplementary material (“Estimation of walking speed and knee flexion angle predictor”). Comparison of KJCF prediction accuracy to existing studies is also presented in the Supplementary material.

Compared to the predictor set of our previous study [22], we replaced frontal plane angle with sagittal plane angle in the predictors because we had no well-established way to estimate the frontal plane angle. Furthermore, knee sagittal plane angle may contain information about knee extension moment and the force exerted on the knee joint by knee extensors at the beginning of the stance phase. A recent study by Saiki et al. [31] showed that OpenPose can estimate even the radiographic hip-knee-ankle angle well; thus, future research could utilize OpenPose to retrieve knee angles in both the sagittal and the frontal planes for the predictor set. In the case of the frontal plane, a single image from a static standing pose would suffice.

Because we showed that the time series of the knee joint flexion angle can be obtained outside the motion laboratory, future research could investigate if such time series information obtained outside the motion laboratory could be utilized for predicting entire KJCF loading time series, like some existing studies have done with MOCAP data [2, 16]. Predicting the entire KJCF loading time series could enable the calculation of parameters such as loading impulses and their utilization in predictive models of knee degeneration [25] in addition to loading peaks. Moreover, the fact that our models predict only KJCF peaks is an important limitation. For out-of-laboratory uses, other information such as joint moments could be useful in addition to KJCFs [35]. Therefore, while our methods cannot replace full musculoskeletal simulation, they may prove useful in cases where only a limited set of musculoskeletal simulation outputs is required. However, the analysis pipeline we present could also be used to train models for predicting, e.g., joint moment peaks. In their current state, our models could be used, e.g., to evaluate whether changing the weight, walking speed, or knee flexion angle during stance results in increased or decreased knee joint contact force maxima; this sort of directional accuracy has been presented as one simple metric relevant for out-of-laboratory use [21]. Although our previous work [22] reported KJCF peak prediction accuracies similar to those in this study and noted that the accuracies were on the same scale as the inherent variability of KJCF peaks during repeated walking trials, actually using our models in out-of-laboratory applications remains to be demonstrated in future studies.

## Conclusions

We trained ANNs to predict KJCF peaks with simple predictors that can be obtained without MOCAP. We demonstrated IMUs and VCs as alternative modalities to MOCAP, but our trained ANNs do not require any specific modality. This modularity means that the ANNs could be used for predicting KJCF peaks with predictors estimated from other modalities than IMUs or VCs (e.g., stopwatch or photocells for walking speed and electrogoniometers for KFA), or with the same modalities from different manufacturers. The future use of stopwatches or photocells is further motivated by our observation that the KFA predictor appears to be unimportant when walking speed is present in the predictor set. Hence, if we dropped the KFA predictor but otherwise kept the predictor set unchanged, we could utilize even simpler measurement technologies than we did in the present study. However, the usability of only KJCF peaks as the output is a limitation for out-of-laboratory applications.

## Supplementary Information

Below is the link to the electronic supplementary material.

Supplementary file 1 (PDF 1767 kb)

Supplementary file 2 (AVI 220796 KB)
