# Supplementary material for: Predicting Knee Joint Contact Force Peaks During Gait Using a Video Camera or Wearable Sensors
Source: Ann Biomed Eng. 2024 Aug 3;52(12):3280–94. doi: 10.1007/s10439-024-03594-x (PMC11561138; doi:10.1007/s10439-024-03594-x)
Supplement: Supplementary file 1 — (PDF 1767 kb) [file 10439_2024_3594_MOESM1_ESM.pdf]

Supplementary material: Predicting knee joint contact force peaks during gait using a video camera or wearable sensors

Supplementary methods for video camera data

Correcting for issues in OpenPose keypoint detection and assignment to people other than the participant

During the measurements, the operator of the measurement equipment sat at a computer in the background of the video feed. OpenPose had no way of knowing which person (the operator or the participant) was the person to track, so it detected the keypoints of both people. The operator was detected by OpenPose even when a physical screen covered most of the operator. The keypoints belonging to the participant were identified by comparing the lengths of segments between keypoints; because the operator was in the background and sitting, the lengths of the operator's segments were shorter. However, sometimes some keypoints were erroneously assigned to the wrong person when the participant occluded the operator in the image (e.g., the left leg of the operator was erroneously assigned to belong to the participant). These occasions were rare and resulted in the erroneous assignment of keypoints for one or just a few frames and were filtered out in later analysis steps. However, because filtering cannot completely eliminate their effect on the data, they may have affected the results slightly.

Correction of camera parallax

A displacement of one pixel near the edges of the field of view (FOV) of the camera represents more real-world displacement than a displacement of one pixel at the center of the FOV. Therefore, the trajectories of OpenPose keypoints must be modulated as a function of how distant they are from the center of the FOV. To account for camera parallax, we first applied an offset to them equal to half the resolution of the image. This centered the trajectories so that the middle of the image, instead of the upper left corner, became the origin. Next, for each keypoint trajectory in each frame, we calculated the angle from the center of the image as

$$\alpha = \arctan\left(2 \times \mathrm{POS} \times \frac{\tan\left(\frac{\mathrm{FOV}}{2}\right)}{\mathrm{RES}}\right), \qquad (1)$$

where $\times$ is the scalar multiplication operator, α is the angle from the center of the image to the keypoint when viewed from the camera, FOV is the field of view of the camera in degrees, POS is

the position of the keypoint in the image along one dimension, and RES is the resolution of the image. The equation follows from trigonometry as illustrated in **Figure S1**.

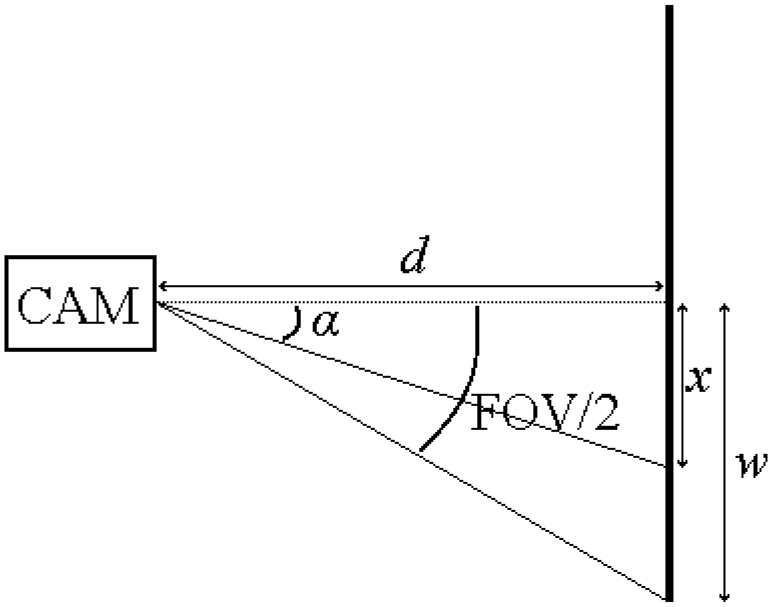

***Figure S1**. Illustration of the geometry of the field of view.*

For an angle from the center of the image to an arbitrary keypoint,

$$\tan(\alpha) = \frac{x}{d},$$

where α is the angle, x is the distance between the keypoint and the center of the image along the x-axis, and d is the distance between the camera and the plane where the motion occurs. Therefore, the angle equals

$$\alpha = \arctan\left(\frac{x}{d}\right). \qquad (2)$$

If the keypoint is located at the edge of the image, we have

$$\tan\left(\frac{\text{FOV}}{2}\right) = \frac{w}{d},$$

where w is half the resolution of the image. Solving for d, we have

$$d = \frac{w}{\tan\left(\frac{FOV}{2}\right)}. \qquad (3)$$

And inserting (3) into (2) we get

$$\alpha = \arctan(x \times \frac{\tan\left(\frac{FOV}{2}\right)}{w},$$

which equals (1) because w is half the resolution of the image.

Estimating walking speed from video camera data

To estimate the walking speed of the participant from video camera data, we made assumptions about the participant's height in relation to the distances between different keypoints detected by OpenPose and tracked the horizontal trajectory of the mid-hip keypoint. First, we calculated the linked height of the participant as the distance along the path traveling in straight lines through the nose, neck, mid-hip, hip, knee, ankle, and heel keypoints, in that order. We then assumed that the linked height equaled 1.2 times the actual height of the participant. Now we could formulate the meters-per-image-pixel factor (MPIP) as

$$\text{MPIP} = 1.2 \times \frac{\text{ACTUAL HEIGHT [m]}}{\text{LINKED HEIGHT [pixels]}}.$$

To calculate walking speed, we first calculated the absolute horizontal speed of the mid-hip keypoint in consecutive frames. We calculated the mean of the framewise speeds, given in pixels per frame, and multiplied it with the MPIP factor to obtain the walking speed as meters per frame. Finally, we multiplied the result with the sampling frequency of the video camera, given in frames per second, to obtain the walking speed in meters per second.

Estimating knee flexion angle from video camera data

To estimate knee flexion angle for each frame in the video data, we calculated two vectors for the leg: a thigh vector $\hat{t}$ from the hip keypoint to the knee keypoint, and a shank vector $\hat{s}$ from the knee keypoint to the ankle keypoint (**Figure S2**). We then normalized the vectors and calculated the angle between them using the four-quadrant inverse tangent through MATLAB's atan2d function:

$$\text{angle} = \text{atan2d}(t_x s_y - t_y s_x, t_x s_x + t_y s_y)$$

where t_x and t_y and s_x and s_y denote the x and y components of the thigh and shank vectors, respectively.

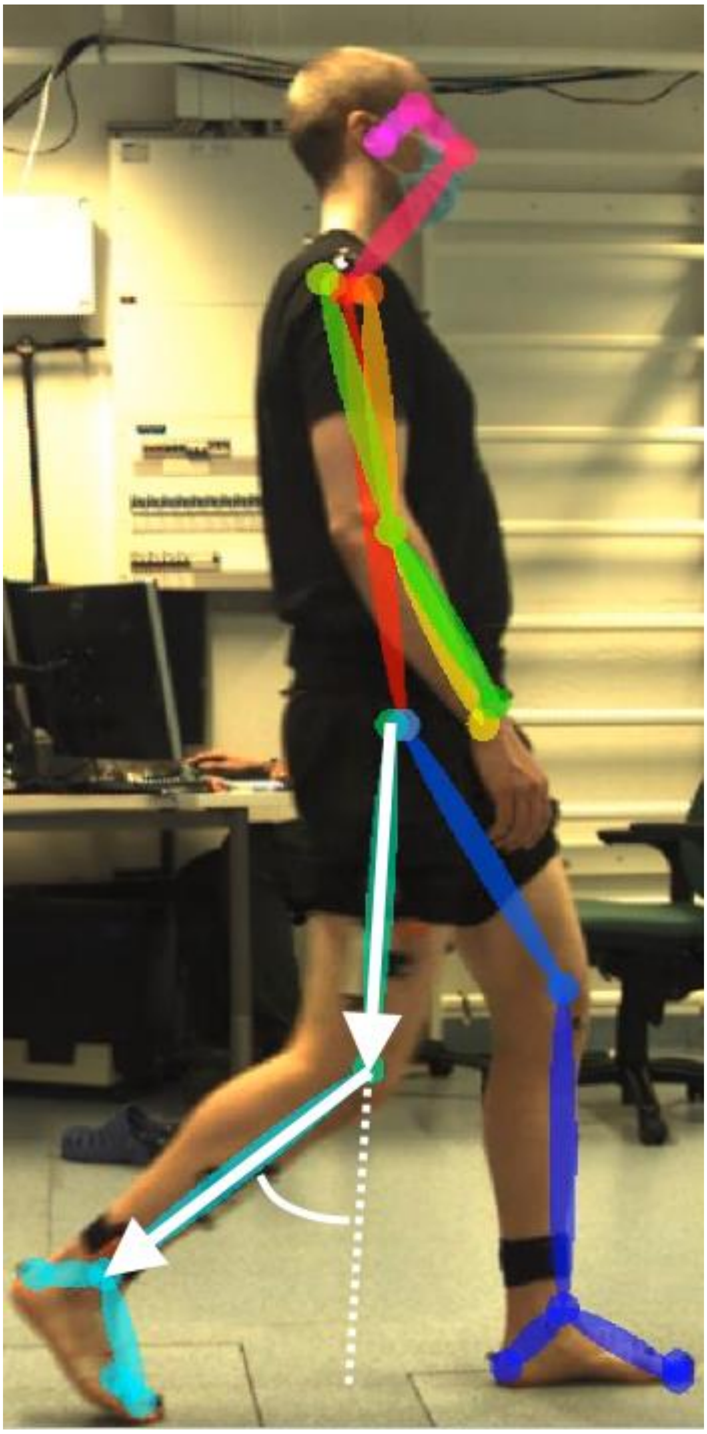

Figure S2. *A single frame from the video data of a walking trial. Keypoints detected by OpenPose are overlaid on the image with colored circles. The white solid arrows denote the thigh and shank vectors that we calculated. The curved white line denotes the knee flexion angle between the two vectors.*

Supplementary methods for inertial measurement unit data

Estimating walking speed from IMU data

We retrieved 3D orientation data from the thigh and shank IMUs on both legs. We modelled the thighs and shanks as rigid bodies and made the following assumptions:

- The length of the thigh is height/3.7.
- The length of the shank is height/3.5.
- The proximal ends of the thighs are connected.
- In each leg, the thigh and shank are connected.
- At the first frame of the data, the thighs and shanks are similarly oriented.

We retrieved the thigh and shank scale factors of the first two assumptions by performing a grid search on a subset of the data and finding the factors that minimized the error between IMU-estimated and MOCAP-based walking speed. The third assumption ignores the pelvis and approximates hip joint centers to align on the sagittal plane, while the fourth assumption models the knees as ball joints; although biomechanically invalid, these two assumptions allow great simplification at a small accuracy cost. The last assumption was supported by the fact that all walking trials started with the participant in a static standing pose.

Using the orientation data from IMUs at each frame, we rotated the thighs and shanks and calculated the distance between the distal ends of the shanks. We then calculated the distance between the distal ends of the shanks at each frame as

$$d = \sqrt{(x_l - x_r)^2 + (y_l - y_r)^2 + (z_l - z_r)^2},$$

where d is the distance between the distal ends of the shanks (i.e., distance between feet), x, y and z are the triaxial components of positions of the distal ends of the shanks, and subscript indices indicate left (l) or right (r) leg.

We identified the two midmost peaks of the resulting distance time series and calculated stride length as the sum of those peaks (**Figure S2**, the peak denoted by solid vertical line and the immediately following peak). Stride duration was calculated as the time that passed between the peaks immediately adjacent to the midmost peak (**Figure S2**, the peaks marked by two dashed vertical lines). Finally, we calculated walking speed by dividing stride length by stride duration.

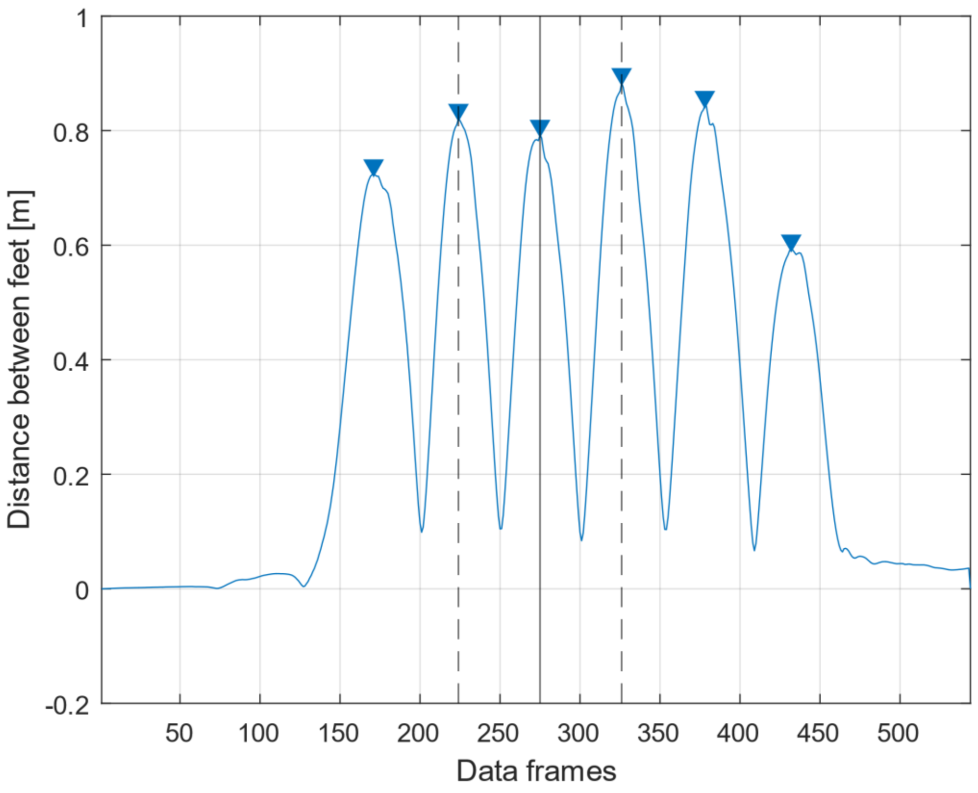

Figure S3. *An example of the time series of distance between the distal ends of the shanks. Identified peaks, where the feet are assumed to be maximally distant, are marked with blue upside-down triangles. The solid vertical line marks the midmost peak and the dashed vertical lines the peaks immediately adjacent to it. Stride length was calculated by summing the midmost peak and the following adjacent peak. Stride duration was calculated as the time between the peaks marked by dashed vertical lines (i.e., the difference in data frames divided by IMU sample rate).*

Note that when finding the thigh and shank scale factors with grid search, the MOCAP-based walking speed was measured only during the stance phase. Therefore, the thigh and shank lengths in our model are optimized to estimate walking speed during the stance phase, not necessarily over a full gait cycle, even though our physical model uses the full gait cycle to calculate stride length. For our purposes, estimating walking speed during stance phase is better than estimating walking speed over a full gait cycle because the ANNs that the walking speed estimates are fed to are trained on walking speeds from the stance phase; however, if others were to use our walking speed estimation model simply to estimate walking speed during the full gait cycle, they should run grid search for thigh and shank lengths against MOCAP-based walking speeds from the full gait cycle.

Estimating knee flexion angle from IMU data

We retrieved 3D orientation data from the thigh and shank IMUs on the leg of interest and assumed that the thigh and shank are similarly oriented at the first frame of data. We then converted the rotation matrix orientations of the IMUs to axis-angle representations. The axis-angle representation

of a rotation is a 3D vector whose direction describes the axis of rotation and magnitude the amount of rotation.

We conducted principal component analysis on the axis-angle representations to find the axes of greatest rotational variance in both IMUs. We assumed that during gait, the IMUs on the femur and tibia mostly rotate on the sagittal plane. Therefore, we assumed that the first principal components of the axis-angle representations represented motion in the sagittal plane, i.e., motion effectuated by flexion and extension of the hip and knee. Because of our earlier assumption of identical orientation at first frame, we interpreted differences in the first principal components between thigh and shank IMUs as knee flexion or extension.

To distinguish between flexion and extension, we assumed that during walking, the knee has a greater maximal flexion angle than maximal extension angle, and we flipped the sign of the resulting knee flexion time series if it showed greater maximal extension than maximal flexion. For each trial, the sign-corrected time series was then retrieved as the IMU-estimated knee flexion angle.

Our assumption regarding the first principal component of rotation only holds when the participant walks a straight line without turning. If the participants turns, the rotation around the vertical axis may be greater than the rotation around the flexion-extension direction of the knee, replacing the first principal component of rotation. This effect could be avoided by calculating the principal components from the rotation between the two IMUs rather than for each IMU separately. However, it would have enabled a new source of error if the axes of the IMUs are defined differently. To define their nonvertical axes, our IMUs use magnetic fields, which may be differently present on thigh and shank level and cause misalignment in the IMUs' nonvertical axes. This misalignment would show as an error in the measured rotation between the two IMUs, which is why we calculated the principal components for each IMU before calculating the difference in rotation between the two IMUs.

Details of our artificial neural networks

The ANNs used the Levenberg-Marquardt backpropagation training algorithm with Bayesian regularization (MATLAB’s trainbr function). The input layer comprised six nodes corresponding to the predictors and the input data underwent minmax mapping to the range [-1, 1]; this was a recommended procedure when using Bayesian regularization in the training. In the hidden layer, we had only a single hidden node and it utilized a hyperbolic tangent activation function. The use of a

single node was justified because our predictors were very general and unlikely to describe complex relationships with the output. Finally, a hyperbolic tangent activation function fit our purpose well because we only had a single hidden layer and therefore the vanishing gradient problem, which is common in deep neural networks, was unlikely to occur and necessitate the use of a ReLU or a similar non-saturating activation function [7]. The output layer then simply used the dot product of the weights and the output of the hidden node, and finally, summed the output layer bias to it.

We trained the ANNs by first pre-training them using the CAROT data [1] and then continuing training, i.e., fine-tuning the ANNs, with the data from the four open datasets [4,6,8,15], but otherwise similarly as in our previous work [9]. We used CAROT data to pre-train the neural networks because the CAROT dataset contained knee osteoarthritis patients and we wanted to fine-tune the network with healthy subjects from the four open datasets because we would be using the ANNs to predict KJCF peaks of healthy participants. Therefore, we finally obtained nine different trained ANNs to predict loading response, terminal extension, and maximum peak over the sum of compartmental loadings, medial compartmental loading, and lateral compartmental loading.

Elimination of invalid trials from the test set

When constructing the independent test set, some walking trials had to be eliminated and thus the number of valid trials that could be used for quantifying the ANNs varied between different participants. Reasons for trial elimination included participants stepping outside or only partially on the force plate, measurement-related artifacts in the data (see the supplements of our previous study[9] for examples), and participant incompliance with instructions during some walking trials leading to data where some analysis steps could not be used because the underlying assumptions failed to apply. In our previous study[9], this elimination was done by visually inspecting KJCF time series, but in this study, we instead did the elimination by visually inspecting GRF time series and time series of solved hip and knee angles for anomalies. This change was done to avoid possible unconscious bias in favoring certain types of KJCF time series. Furthermore, some trials were automatically eliminated because the KJCF peak from musculoskeletal simulations could not be identified (e.g., in slow instructed velocity trials where the KJCF time series sometimes had no distinct loading response peak).

Supplementary summarizing information of the collected dataset

Mean knee flexion angle and standard deviation show that as instructed walking speed increases among the participants in the collected dataset, the knee flexion angle maximum during the first half of the time series increases (**Figure S4**).

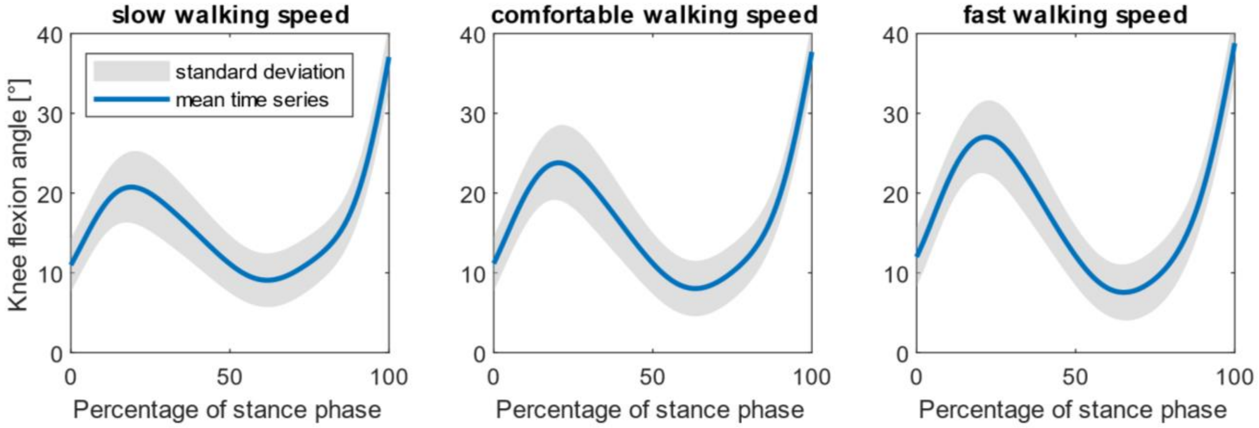

Figure S4. *Mean knee flexion angle time series (blue line) and standard deviation (shaded area) of participants in the collected dataset. The knee flexion angle is calculated as the inter-subject mean of intra-subject mean knee flexion angles. The standard deviation is calculated as the inter-subject standard deviation of intra-subject mean knee flexion angles.*

Mean KJCF time series and standard deviation show that if the participant walks at slower-than-comfortable walking speed, the loading response peak decreases and becomes less distinct, and if the participant walks at faster-than-comfortable walking speed, the loading response peak increases and becomes more distinct (**Figure S5**).

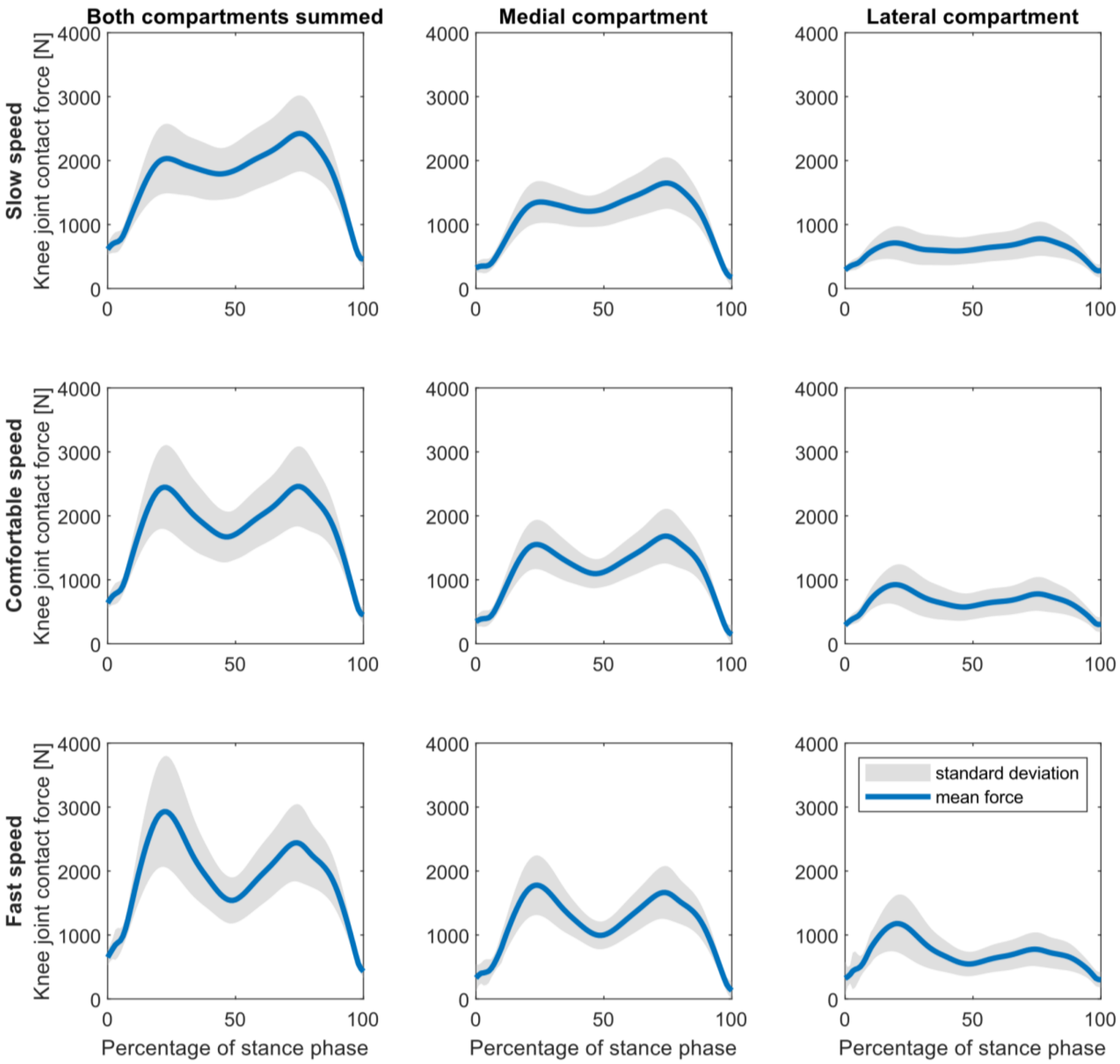

Figure S5. *Mean knee joint contact force time series (blue line) and standard deviation (shaded area) of participants in the collected dataset. The knee joint contact force is calculated as the inter-subject mean of intra-subject mean knee joint contact forces. The standard deviation is calculated as the inter-subject standard deviation of intra-subject mean knee joint contact forces. The mean curves are presented for medial (center column) and lateral (right column) compartments and both compartments summed (left column); and for slow (first row), comfortable (second row), and fast (third row) instructed walking speeds.*

Supplementary fitted multiple linear regression equations

The fitted multiple linear regression models were of the form

$$y = a * \text{mass} + b * \text{height} + c * \text{age} + d * \text{sex} + e * \text{walking speed} + f * \text{KFA predictor} + g$$

where mass is given in kg, height in mm, age in years, sex as {1=male, 0=female}, walking speed in m/s, and the KFA predictor in degrees. Coefficient g is the intercept. The fitted coefficients *a* through *g* for different response variables are presented in **Table S1**.

Table S1. *Coefficients of fitted first-order multiple linear regression models for predicting KJCF peaks. Coefficients a, b, c, d, e, f, and g are the weights of mass, height, age, sex, walking speed, and the knee flexion angle predictor, respectively.*

Response variable	a	b	c	d	e	f	g
**full-stance max (summed)**	28.39	-0.23	-3.58	167.71	599.05	29.74	-417.13
**full-stance max (medial)**	19.49	-0.20	-2.25	108.91	451.46	15.16	-146.49
**full-stance max (lateral)**	10.07	-0.02	-0.36	48.97	184.87	15.93	-394.16
**loading response (summed)**	23.13	-0.05	-2.89	22.99	443.75	58.79	-901.12
**loading response (medial)**	14.95	0.00	-1.70	2.51	300.19	29.10	-406.87
**loading response (lateral)**	9.49	0.01	0.64	0.66	196.46	25.26	-689.50
**terminal extension (summed)**	26.60	-0.19	-3.79	202.77	540.23	6.63	-89.12
**terminal extension (medial)**	18.99	-0.17	-2.47	125.36	390.90	7.29	-35.48
**terminal extension (lateral)**	8.19	-0.02	-1.26	72.67	178.58	-1.13	-84.45

Supplementary KJCF prediction results

Effect of instructed walking speed on KJCF prediction accuracy

For baseline and both portable modalities, the predicted KJCF peaks had the smallest NMAE when the participants walked at their comfortable speed, except for medial terminal extension peaks where slow walking speed resulted in slightly smaller NMAE (**Figure S6**). However, for most response variables, the greatest NMAE occurred when the participant walked at slow speed.

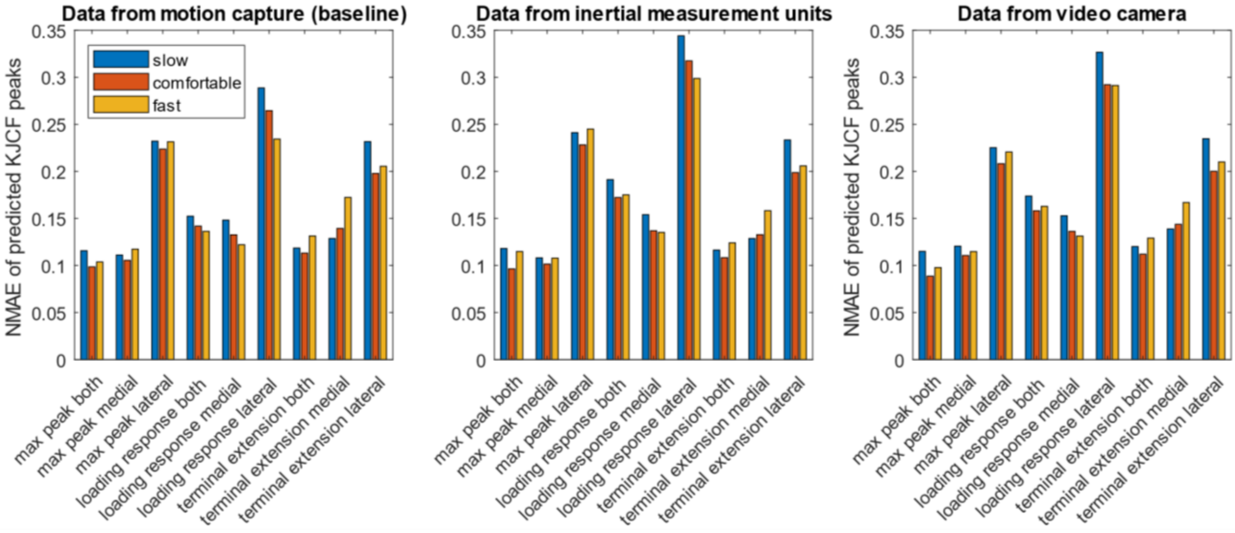

***Figure S6**. Effect of instructed walking speed on the prediction accuracy of knee joint contact force (KJCF) peaks. The bar charts show the mean absolute errors normalized to the mean of response values (NMAE) of predicted KJCF peaks for different response variables (on x-axis). The walking speed and knee flexion angle predictors are estimated from inertial measurement unit data (left) and video camera data (right). "Slow" indicates instructed walking speed that is 25% slower than comfortable walking speed, and "fast" 25% faster than comfortable walking speed.*

Effect of absolute walking speed on KJCF prediction accuracy

Between the absolute error of KJCF peaks and absolute walking speed, we observed statistically significant weak correlation ($0.1<R<0.3$, $p<0.05$) for all modalities and response variables except for lateral terminal extension peaks (**Figure S7**). For lateral terminal extension peaks, statistically

significant very weak correlation (R=0.06) was observed when walking speed and KFA predictor were estimated from video camera data, but not otherwise.

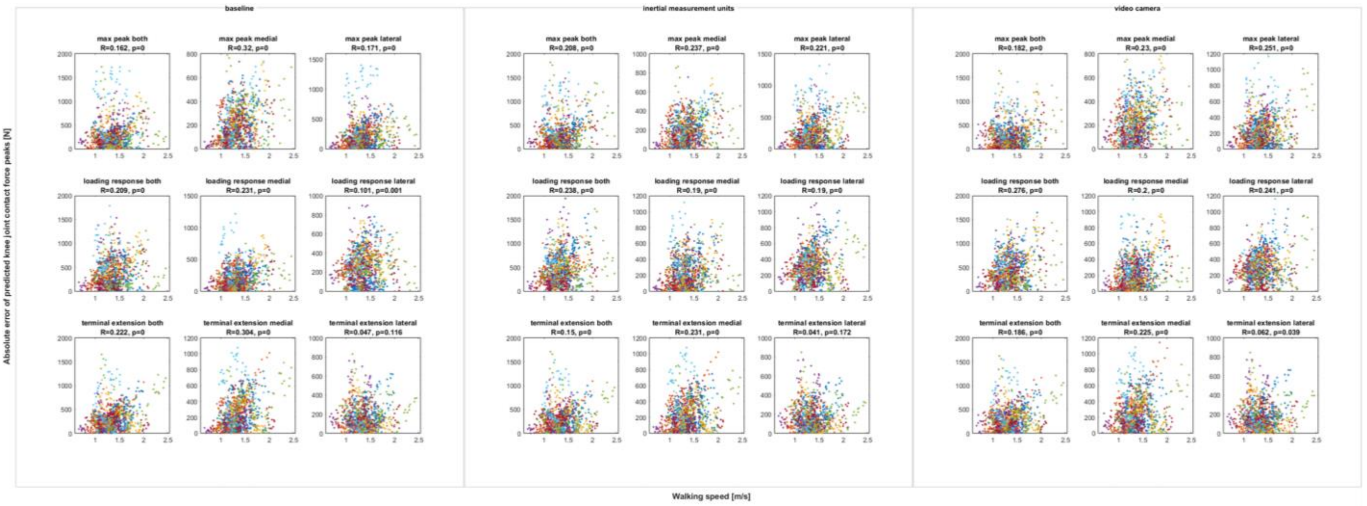

***Figure S7**. The effect of absolute walking speed on the prediction accuracy of knee joint contact force (KJCF) peaks, with the prediction accuracy of each response variable shown in its own subplot. Data points of one color belong to the same participant. Predictions are made with walking speed and knee flexion predictor from motion capture (left), inertial measurement unit (center), and video (right) data.*

Scatter plots of predicted peaks versus reference peaks for IMU and VC data

The regression plots of predicted peaks from IMU data (**Figure S8**) and VC data (**Figure S9**) with respect to reference values from musculoskeletal simulations are similar to the plot of predicted peaks from baseline (motion capture) data with respect to reference values (**Figure 5** in the main manuscript).

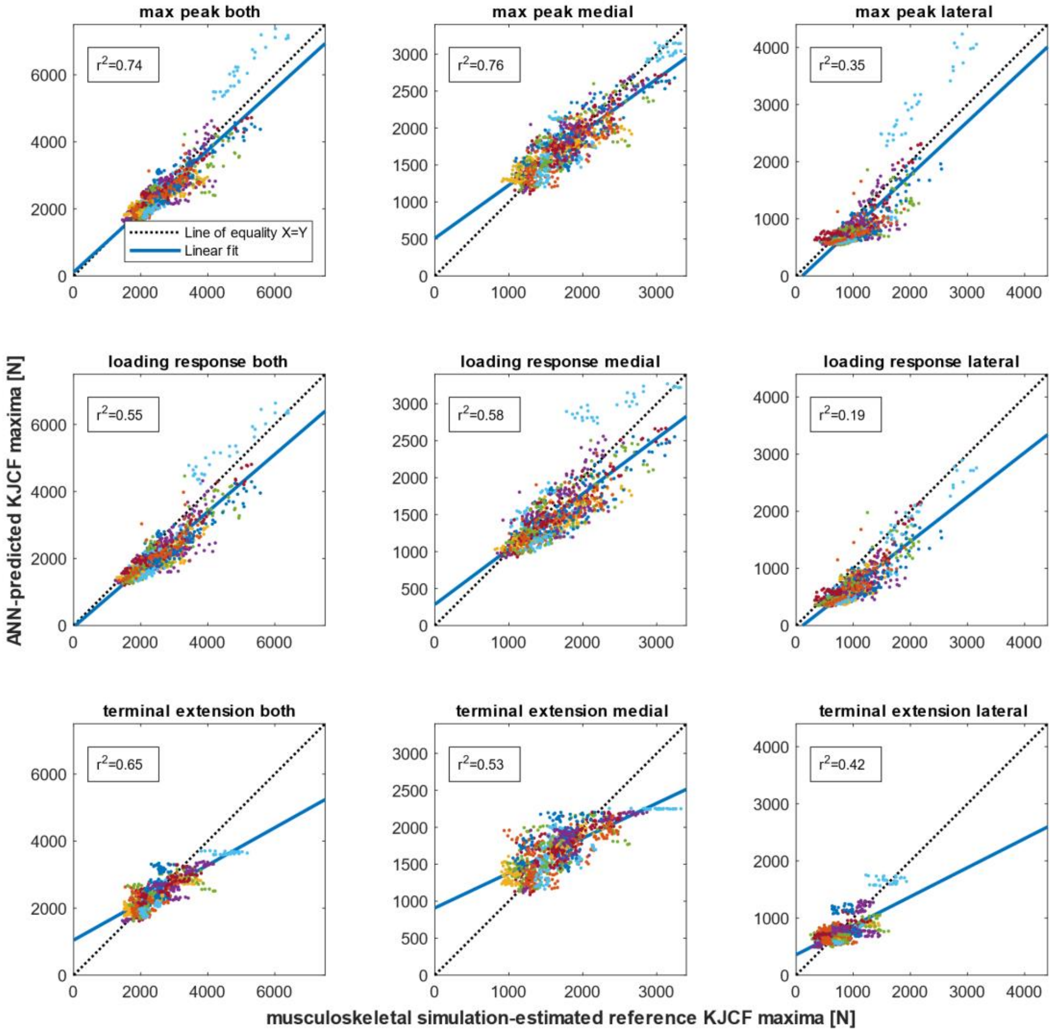

Figure S8. *Accuracies of predicting KJCF peaks with ANNs when utilizing walking speed and knee flexion angle from inertial measurement unit data. Each point represents a KJCF peak from one walking trial, calculated with musculoskeletal simulation and modeling (x-axis) and ANNs (y-axis). Walking trials from different subjects have different colors. All plots use the same participants and walking trials.*

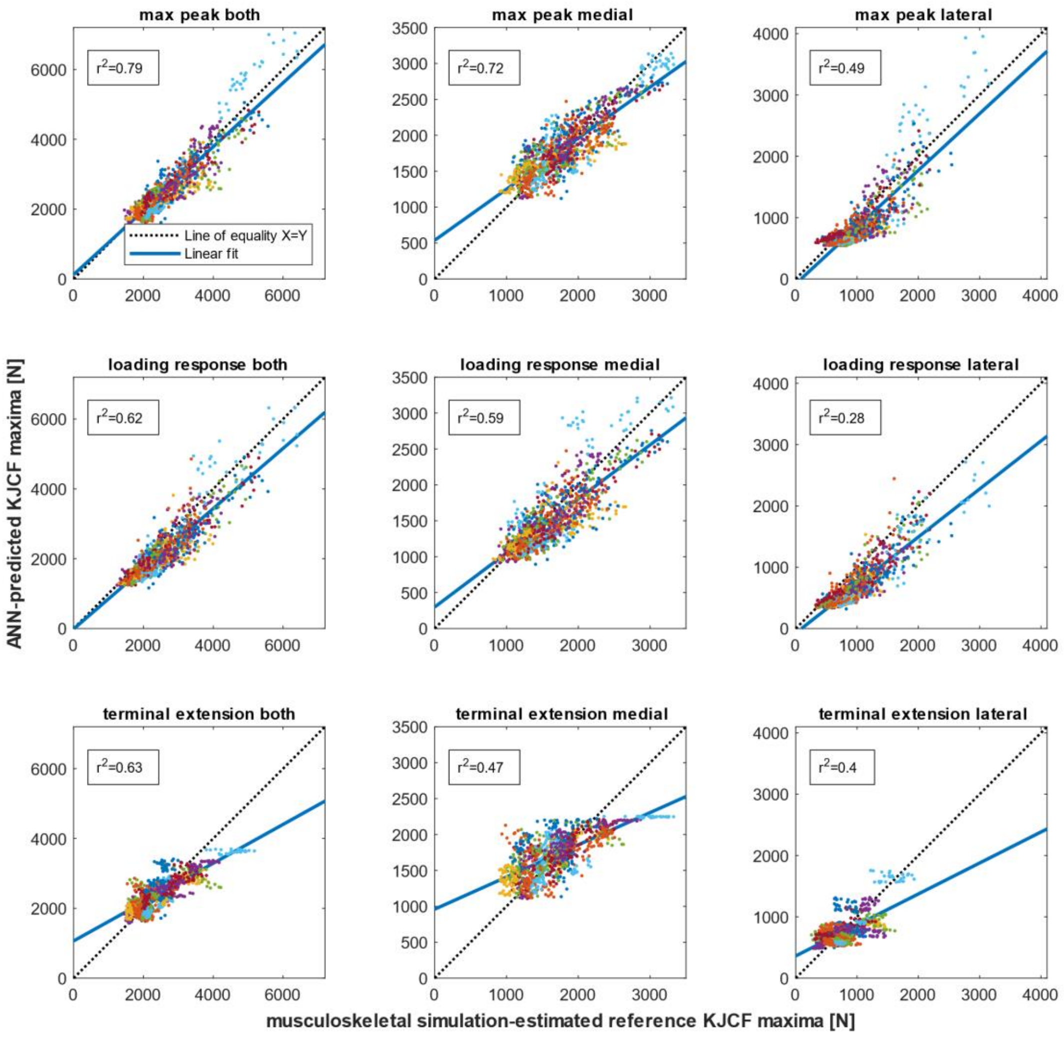

Figure S9. *Accuracies of predicting KJCF peaks with ANNs when utilizing walking speed and knee flexion angle from video camera data. Each point represents a KJCF peak from one walking trial, calculated with musculoskeletal simulation and modeling (x-axis) and ANNs (y-axis). Walking trials from different subjects have different colors. All plots use the same participants and walking trials.*

Supplementary discussion

Accuracy of predicting KJCF peaks

The fact that slow instructed velocity often resulted in the worst prediction accuracy of KJCF peaks (**Figure S6**) may seem odd because at slow walking speed, there should be minimal motion of the soft tissue that could generate artefacts in IMU data and little motion blur to generate noise in VC data. We suspect that the reason for the relatively high prediction error is the fact that at slow

walking speeds, the KJCF peaks are less distinct than with comfortable and fast walking speeds. This lack of distinction makes their identification difficult, slightly reducing the number of data points available for training the ANNs. Although absolute walking speed correlated weakly with the prediction error of KJCF peaks (**Figure S7**), walking trials with a broader range of absolute walking speed are required to draw a clear conclusion about the effect of absolute walking speed on prediction accuracy because especially walking speeds above 2 m/s had a limited number of data points.

In this work, we defined the response variables by assuming that the knee joint loading time series has a double-bump profile. This assumption might be unrealistic for lateral peaks (where there may be more than two peaks), for loading peaks of pathologic subjects (where, e.g., the first peak may be undetectable and the loading curve has only the terminal extension peak), or for very slow walking speeds (where the first loading peak may also be undetectable). Failure to detect the assumed peaks leads to elimination of the trial, so redefining the response variables and improving the peak detection algorithm could increase the number of trials available for training and the variety of the training samples, which could improve prediction accuracy and result in more robust trained networks for predicting KJCF peaks. Hence, future studies should investigate more robust ways to identify the loading peaks.

Finally, we intentionally evaluated each walking trial in the test set individually instead of averaging several trials and extracting mean predictors and mean ground truth KJCF peaks for each subject. While averaging trials could have improved results (and ensured that data points are independent from each other as is usually assumed in correlation analysis), we opted against doing so, as in our conceptualized out-of-laboratory application it seems more feasible to record one or only a few trials rather than many. Furthermore, a recent study [5] indicated that at least in running, it would take a 2-digit number of trials to reach a mean that is mostly unvarying. The study also found that among joint angles in running, peak knee flexion is particularly susceptible to strong variation between repeated trials. It seems possible to us that similar effects could be observed in walking. Therefore, we used individual trials to capture per-trial variation and remain close to the conditions of the conceptualized out-of-laboratory application, although it would certainly be preferable if the loading peaks from several trials were averaged in an out-of-laboratory setting to obtain a reliable mean loading estimate.

Comparison of KJCF prediction accuracy to existing studies

Comparison of the KJCF peak prediction accuracy of our trained models to existing literature is difficult because few studies directly predict compressive KJCF using machine learning methods. Furthermore, studies utilizing video capture data do not necessarily use the information from video data to train prediction models but may instead use that data as a substitute to marker data and conduct musculoskeletal simulations[20]. In such cases, identifying a KJCF prediction model whose accuracy could be evaluated and used as a comparison becomes difficult.

However, for IMU data, some recent studies directly predict KJCFs providing some information on the accuracy of prediction of KJCF peaks. For instance, Stetter et al. trained an ANN to predict net knee joint force (excluding muscle forces) time series using the signals of two IMUs as input and reported an absolute percentual error of 13.8% for predicting compressive force peaks during walking [17]. In our study, the absolute percentual error for the summed loading peak over the full time series was 10.6%, which is slightly better. In another example, di Raimondo et al. trained a machine learning model for predicting KJCFs from IMU data [13]. For predicting the first (loading response) peak of the medial compartment, lateral compartment, and total loading, they reported mean absolute differences of 0.21 BW, 0.08 BW, and 0.24 BW, respectively. For predicting the second (terminal extension) peak, they similarly reported 0.27 BW, 0.12 BW, and 0.27 BW. Our corresponding mean absolute differences for the first peak were 0.13 BW, 0.32 BW, and 0.17 BW, and for the second peak, our mean absolute differences were 0.15, 0.23, and 0.12 BW. Therefore, our total and medial compartment KJCF peak predictions were more accurate, but our lateral compartment KJCF peak predictions were far less accurate than those reported by di Raimondo et al. While these results for predicting KJCF peaks are encouraging given the fact that our trained models used a very simple predictor set, we note that unlike the methods of Stetter et al. and di Raimondo et al., our method cannot predict entire time series. Additionally, the reader should note that different studies may have used different subject demographics and methods to calculate accuracy metrics, making direct comparisons between studies difficult. Consequently, the comparisons presented here should be considered as only approximate.

Estimation of walking speed and knee flexion angle predictor

Several methods have been proposed in literature to estimate kinematic parameters from IMU data [3,14,19]. The methods proposed in literature include neural networks, which are difficult to explain in simple terms and generalize poorly if the training data is insufficiently representative [18]. In addition

to being easily explainable, which is a requirement in clinical settings [2,16], our methods are independent from IMU coordinate system configurations: if 3D orientation data can be retrieved in some coordinate system, our methods should remain usable. Nonetheless, our methods are based on assumptions that may not hold in gait pathologies. For instance, if gait contains major motion outside the sagittal plane, the axis of greatest rotational variance in thigh and shank may differ from the axis the knee is rotating around. Furthermore, we did not separate an independent subset of the IMU data to find the lengths of the femur and tibia relative to the height of the participant (i.e., partially the same data was used to find femur and tibia lengths that minimize walking speed estimation error and to quantify the error). However, we consider the effect of this decision insignificant when considering that all subjects used the same IMUs – in this case, hardware is probably a greater factor in robustness of the walking speed estimation algorithm than the effect of using partially the same IMU data for finding the lengths of femur and tibia and quantifying walking speed estimation error. Finally, we used orientation data calculated by the Xsens proprietary Kalman filter [11], which may represent accuracy unattainable to some IMUs.

For VC data, we expect the OpenPose algorithm to work with different video cameras because nothing fundamental changes between different devices. OpenPose works robustly with different resolutions, although we noticed that too high resolution will lead to OpenPose falsely detecting human shapes in the background; this is also why we ran OpenPose at a resolution lower than the original resolution of the video data (480x272 versus 1280x720). We view this as an advantage because it shows that the VC-based method may detect keypoints even from low-resolution images; this lowers hardware requirements to perform motion analysis with OpenPose. Additionally, the default BODY_25 model of OpenPose, which we also used, is trained on images labeled by amateurs rather than biomechanics experts, meaning that the keypoints identified by OpenPose are unlikely to be located exactly where the label should anatomically be (e.g., the hip keypoint is unlikely to be exactly in the hip joint center). However, the impact of this error is reduced when we are interested in tracking the displacement of anatomical landmarks across the image (when estimating walking speed) or drawing vectors between them to represent limbs (when estimating knee flexion angle) rather than their exact positions. Finally, because human pose estimation algorithms usually produce positions and confidences of anatomical keypoints similarly to OpenPose, other available human pose estimation methods[10] should be compatible with our method to estimate walking speed and knee flexion angle from video data. This is advantageous in the context of using the method in clinical settings, as clinics may have limitations on what software they can use.

Therefore, although we developed methods to estimate walking speed and knee flexion angle using laboratory-free setups and analyzed some accuracy measures for them, the scope of our manuscript was not to conclusively validate such methods but simply to demonstrate that they have potential for estimating useful predictors. Our methods to estimate knee flexion angle and walking speed should be viewed as instructions for one way to handle their estimation. Future studies could investigate whether those instructions work for other IMU manufacturers and different algorithms to estimate IMU orientation, and whether human pose estimation algorithms other than OpenPose can effortlessly be plugged in its place.

The use of IMUs led to elimination of slightly more participants than the use of VCs (four to six depending on response variable; one to two more than VCs). In the case of IMUs, this was often caused by us failing to estimate knee flexion angle properly because the participant, instead of stopping after walking a straight line and standing still, turned to look at the operator of the equipment or turned and started walking back to the starting position before the walking trial was concluded. Resultingly, their IMUs experienced significant rotation around the vertical axis and the first principal component of rotation (see **Supplementary material**, "Estimating knee flexion angle of IMU data") of their thigh and shank IMUs were no longer aligned with the flexion-extension direction, but around the vertical axis. Nonetheless, even in the case of VCs, where the aforementioned failed assumption was never present, two to five participants (depending on response variable) did not have at least five valid walking trials in each configuration because the response variable peaks could not always be successfully identified, and consequently, those trials were eliminated. In the context of possible clinical use in future, the methods will have to be refined to maximize the amount of successful walking trials and to avoid wasting appointment time on discarded measurements.

We presented methods for predicting KJCF peaks from IMU and VC data separately. Both data could also be fused together to complement each other and calculate kinematics more accurately. For instance, Pearl et al. [12] noted that kinematics calculated using constrained fusion of data from Xsens IMUs and keypoints was more accurate than kinematics calculated from the two modalities separately. They noted that IMUs were better at measuring joint flexion, while computer vision methods outperformed the IMUs in measuring joint center positions. In line with the findings of Pearl et al., we also noticed that estimated knee flexion angle from IMU data had smaller error than that from VC data. Therefore, combining the two methods could improve the accuracy of our estimated predictors and the final predicted KJCF peaks. However, we avoided fusion of the

modalities to keep our methods as modular and simple as possible because simplicity and intuitiveness are important criteria when considering the suitability of methods for clinical use.

Considerations when comparing the results to our previous study

The results of this study can be compared to those of our previous study[9] to get an idea of the change in prediction accuracy, but the reader should note that the quantitative results are not directly comparable. This is not only because of different predictors (here, KFA replaced knee frontal plane alignment), but for the following three reasons as well.

First, the neural network training scheme is different. In our previous study, we used 5-fold cross-validation where each fold was in turn used as the test subset and the other folds for training and validation subsets. While no trials from the same subject were allowed to be present in different subsets, dataset overlap was allowed. In the present study, there was no validation subset because we used a training algorithm that stopped training before overfitting based on the values in the weights of the network (see **Supplementary material**, "Details of our artificial neural networks"). Additionally, instead of using k-fold cross-validation we collected a separate test subset. This test subset was completely independent of the training set because we allowed no subject or dataset overlap. Furthermore, the test subset was collected in a different motion laboratory and included different participants, different marker sets etc. than the training subset, further eliminating the possibility of data leakage. Finally, the training subset of this study has more data than that of our previous study.

Second, we performed the exclusion of trials with invalid data differently. In our previous study, it was done based on visual inspection of the KJCF curves retrieved from the MS analysis pipeline. In the present study, we did it by visually inspecting the time series of GRF and hip and knee angles (see **Supplementary material**, "Elimination of invalid trials from the test set"). The method of the previous study risked more bias because the analyst saw the KJCF curves from which the response variables (KJCF peaks) were extracted, and therefore, the analyst's subjective views on validity of KJCF curves may have affected the final valid data. In this study, the analyst's personal views on validity of GRF and joint angles may still have affected the selection process, but likely with a smaller impact on the final KJCF peaks because the KJCF time series themselves were not shown.

Third, there were some differences in the MS analysis pipeline between the studies. In this study, the training subset was analyzed with the MS analysis pipeline described in our previous study and

the test subset with some changes in model scaling (see **Methods**, "Musculoskeletal analysis of the collected dataset").

All these reasons enforce the independence of the test subset from the training subset in this study. In the context of comparing the results, similar prediction accuracy metrics between our previous study and this study means that the generalization accuracy of the networks in this study is at least as good as that of the networks in our previous study because in this study, there are fewer shared latent features between the training and the test subset.

Demographic observations in the training and test sets

In the training set, the BMI of subjects varied between 17.2 kg/m^2 and 51.0 kg/m^2 (as reported in Table 1 of our previous study [9]). The test set we collected contained BMIs between 18.8 kg/m^2 and 40.4 kg/m^2. Additionally, the age of subjects in the training set varied between 19 years and 84 years, but only between 20 years and 45 years in the test set. In terms of the variability of demographic predictors, the test set can be interpreted as a subset of the training set. Therefore, the prediction accuracy metrics in this paper do not necessarily represent the accuracy of predicting knee joint loading for subjects outside the ranges of the demographic predictors in the training set. Consequently, the prediction accuracy metrics may differ from the metrics presented here if the prediction models are used to extrapolate outside the ranges of their trained predictor variables.

In the training set, there were 116 male and 174 female subjects. This ratio was largely affected by the knee osteoarthritis patient dataset [1], where there were 99 female and only 25 male subjects (numbers of subjects in the training dataset is shown in **Figure S10**, where the numbers of knee osteoarthritis patients is highlighted). The test set included 29 male and 17 female participants, of whom 37 (24 male, 13 female) were included while calculating the KJCF peak prediction accuracy metrics. Hence, the presented KJCF prediction models may be biased towards utilizing predictor features more prominently present in female rather than male subjects, while the trained models were tested on a dataset with a male majority. Moreover, because the KOA dataset comprised more than half of the female subjects in the training set and only a fifth of the male subjects[9], the bias introduced in the trained models is likely emphasized when predicting knee joint loading for female subjects. However, we consider the effect of such training bias to be small, because the prediction accuracy metrics on our test set were comparable to the metrics of our previous study [9], where the test sets likely had a similar ratio of male to female subjects as the training set due to the sampling method of the data.

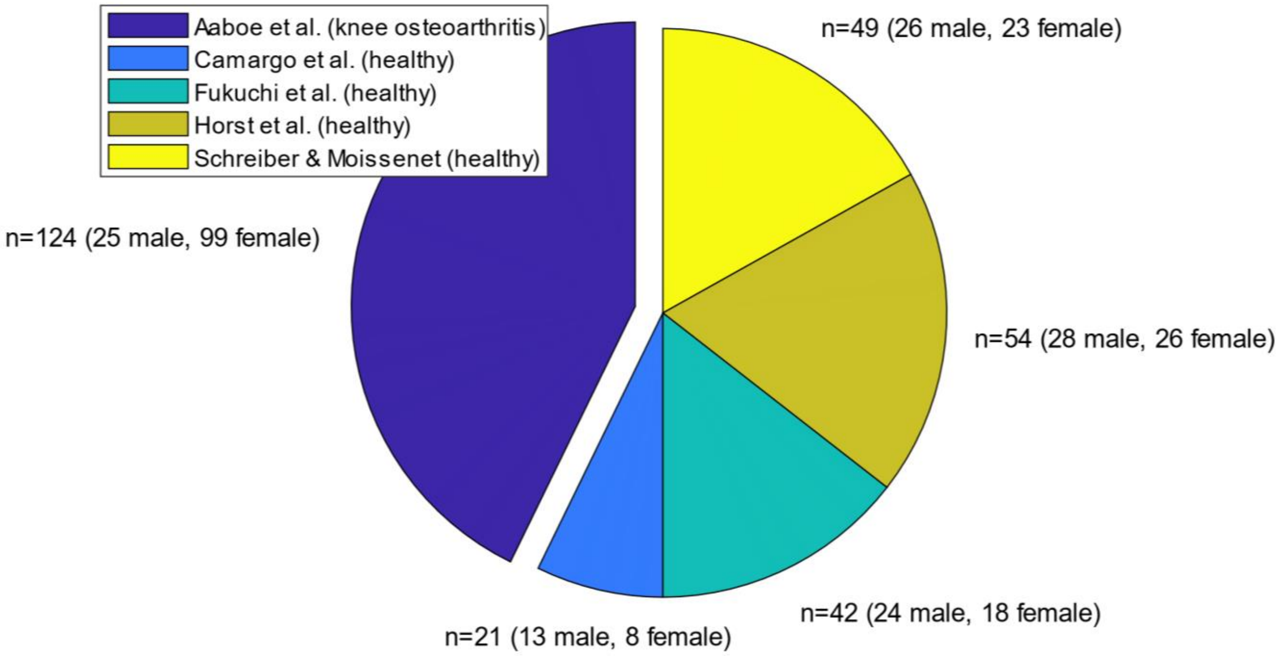

Figure S10. *Distribution of subjects in different datasets that were used for training artificial neural networks. The relatively large dataset containing knee osteoarthritis patients is visually separated from the other datasets containing healthy subjects.*

References

1. Aaboe, J., H. Bliddal, S. P. Messier, T. Alkjær, and M. Henriksen. Effects of an intensive weight loss program on knee joint loading in obese adults with knee osteoarthritis. *Osteoarthritis Cartilage* 19:822–828, 2011.

2. Amann, J., A. Blasimme, E. Vayena, D. Frey, and V. I. Madai. Explainability for artificial intelligence in healthcare: a multidisciplinary perspective. *BMC Med Inform Decis Mak* 20:310, 2020.

3. Al Borno, M., J. O'Day, V. Ibarra, J. Dunne, A. Seth, A. Habib, C. Ong, J. Hicks, S. Uhlrich, and S. Delp. OpenSense: An open-source toolbox for inertial-measurement-unit-based measurement of lower extremity kinematics over long durations. *J Neuroeng Rehabil* 19:, 2022.

4. Camargo, J., A. Ramanathan, W. Flanagan, and A. Young. A comprehensive, open-source dataset of lower limb biomechanics in multiple conditions of stairs, ramps, and level-ground ambulation and transitions. *J Biomech* 119:110320, 2021.

5. Fox, A. S., J. Bonacci, J. Warmenhoven, and M. F. Keast. Measurement error associated with gait cycle selection in treadmill running at various speeds. *PeerJ* 11:e14921, 2023.

6. Fukuchi, C. A., R. K. Fukuchi, and M. Duarte. A public dataset of overground and treadmill walking kinematics and kinetics in healthy individuals. *PeerJ* 2018:1–17, 2018.

7. Goodfellow, I., Y. Bengio, and A. Courville. Deep Learning. MIT Press, 2016.at <http://www.deeplearningbook.org>

8. Horst, F., S. Lapuschkin, W. Samek, K.-R. Müller, and W. I. Schöllhorn. A public dataset of overground walking kinetics and full-body kinematics in healthy adult individuals. *Mendeley Data* , 2019.doi:10.17632/svx74xcrjr.3

9. Lavikainen, J., L. Stenroth, T. Alkjær, P. A. Karjalainen, R. K. Korhonen, and M. E. Mononen. Prediction of Knee Joint Compartmental Loading Maxima Utilizing Simple Subject Characteristics and Neural Networks. *Ann Biomed Eng* , 2023.doi:10.1007/s10439-023-03278-y

10. Needham, L., M. Evans, D. P. Cosker, L. Wade, P. M. McGuigan, J. L. Bilzon, and S. L. Colyer. The accuracy of several pose estimation methods for 3D joint centre localisation. *Sci Rep* 11:20673, 2021.

11. Paulich, M., M. Schepers, N. Rudigkeit, and G. Bellusci. Xsens MTw Awinda: Miniature Wireless Inertial Motion Tracker for Highly Accurate 3D Kinematic Applications. 2018.

12. Pearl, O., S. Shin, A. Godura, S. Bergbreiter, and E. Halilaj. Fusion of video and inertial sensing data via dynamic optimization of a biomechanical model. *J Biomech* 155:111617, 2023.

13. Di Raimondo, G., M. Willems, B. A. Killen, S. Havashinezhadian, K. Turcot, B. Vanwanseele, and I. Jonkers. Peak Tibiofemoral Contact Forces Estimated Using IMU-Based Approaches Are Not Significantly Different from Motion Capture-Based Estimations in Patients with Knee Osteoarthritis. *Sensors* 23:4484, 2023.

14. Ribeiro, P. M. S., A. C. Matos, P. H. Santos, and J. S. Cardoso. Machine Learning Improvements to Human Motion Tracking with IMUs. *Sensors* 20:6383, 2020.

15. Schreiber, C., and F. Moissenet. A multimodal dataset of human gait at different walking speeds established on injury-free adult participants. *Sci Data* 6:1–7, 2019.

16. Slijepcevic, D., F. Horst, S. Lapuschkin, B. Horsak, A.-M. Raberger, A. Kranzl, W. Samek, C. Breiteneder, W. I. Schöllhorn, and M. Zeppelzauer. Explaining Machine Learning Models for Clinical Gait Analysis. *ACM Trans Comput Healthc* 3:1–27, 2022.

17. Stetter, B. J., S. Ringhof, F. C. Krafft, S. Sell, and T. Stein. Estimation of knee joint forces in sport movements using wearable sensors and machine learning. *Sensors (Switzerland)* 19:1–12, 2019.

18. Tan, T., A. A. Gatti, B. Fan, K. G. Shea, S. L. Sherman, S. D. Uhlrich, J. L. Hicks, S. L. Delp, P. B. Shull, and A. S. Chaudhari. A scoping review of portable sensing for out-of-lab anterior cruciate ligament injury prevention and rehabilitation. *NPJ Digit Med* 6:46, 2023.

19. Teufl, W., M. Miezal, B. Taetz, M. Fröhlich, and G. Bleser. Validity of inertial sensor based 3D joint kinematics of static and dynamic sport and physiotherapy specific movements. *PLoS One* 14:e0213064, 2019.

20. Uhlrich, S. D., A. Falisse, Ł. Kidziński, J. Muccini, M. Ko, A. S. Chaudhari, J. L. Hicks, and S. L. Delp. OpenCap: Human movement dynamics from smartphone videos. *PLoS Comput Biol* 19:e1011462, 2023.
